# Measurement of the top quark mass in the $t\bar{t}\rightarrow \text{ lepton+jets }$ and $t\bar{t}\rightarrow \text{ dilepton }$ channels using $\sqrt{s}=7$ ${\mathrm { TeV}}$ ATLAS data

**DOI:** 10.1140/epjc/s10052-015-3544-0

**Published:** 2015-07-17

**Authors:** ATLAS Collaboration, G. Aad, B. Abbott, J. Abdallah, O. Abdinov, R. Aben, M. Abolins, O. S. AbouZeid, H. Abramowicz, H. Abreu, R. Abreu, Y. Abulaiti, B. S. Acharya, L. Adamczyk, D. L. Adams, J. Adelman, S. Adomeit, T. Adye, A. A. Affolder, T. Agatonovic-Jovin, J. A. Aguilar-Saavedra, S. P. Ahlen, F. Ahmadov, G. Aielli, H. Akerstedt, T. P. A. Åkesson, G. Akimoto, A. V. Akimov, G. L. Alberghi, J. Albert, S. Albrand, M. J. Alconada Verzini, M. Aleksa, I. N. Aleksandrov, C. Alexa, G. Alexander, T. Alexopoulos, M. Alhroob, G. Alimonti, L. Alio, J. Alison, S. P. Alkire, B. M. M. Allbrooke, P. P. Allport, A. Aloisio, A. Alonso, F. Alonso, C. Alpigiani, A. Altheimer, B. Alvarez Gonzalez, D. Álvarez Piqueras, M. G. Alviggi, B. T. Amadio, K. Amako, Y. Amaral Coutinho, C. Amelung, D. Amidei, S. P. Amor Dos Santos, A. Amorim, S. Amoroso, N. Amram, G. Amundsen, C. Anastopoulos, L. S. Ancu, N. Andari, T. Andeen, C. F. Anders, G. Anders, J. K. Anders, K. J. Anderson, A. Andreazza, V. Andrei, S. Angelidakis, I. Angelozzi, P. Anger, A. Angerami, F. Anghinolfi, A. V. Anisenkov, N. Anjos, A. Annovi, M. Antonelli, A. Antonov, J. Antos, F. Anulli, M. Aoki, L. Aperio Bella, G. Arabidze, Y. Arai, J. P. Araque, A. T. H. Arce, F. A. Arduh, J-F. Arguin, S. Argyropoulos, M. Arik, A. J. Armbruster, O. Arnaez, V. Arnal, H. Arnold, M. Arratia, O. Arslan, A. Artamonov, G. Artoni, S. Asai, N. Asbah, A. Ashkenazi, B. Åsman, L. Asquith, K. Assamagan, R. Astalos, M. Atkinson, N. B. Atlay, B. Auerbach, K. Augsten, M. Aurousseau, G. Avolio, B. Axen, M. K. Ayoub, G. Azuelos, M. A. Baak, A. E. Baas, C. Bacci, H. Bachacou, K. Bachas, M. Backes, M. Backhaus, E. Badescu, P. Bagiacchi, P. Bagnaia, Y. Bai, T. Bain, J. T. Baines, O. K. Baker, P. Balek, T. Balestri, F. Balli, E. Banas, Sw. Banerjee, A. A. E. Bannoura, H. S. Bansil, L. Barak, S. P. Baranov, E. L. Barberio, D. Barberis, M. Barbero, T. Barillari, M. Barisonzi, T. Barklow, N. Barlow, S. L. Barnes, B. M. Barnett, R. M. Barnett, Z. Barnovska, A. Baroncelli, G. Barone, A. J. Barr, F. Barreiro, J. Barreiro Guimarães da Costa, R. Bartoldus, A. E. Barton, P. Bartos, A. Bassalat, A. Basye, R. L. Bates, S. J. Batista, J. R. Batley, M. Battaglia, M. Bauce, F. Bauer, H. S. Bawa, J. B. Beacham, M. D. Beattie, T. Beau, P. H. Beauchemin, R. Beccherle, P. Bechtle, H. P. Beck, K. Becker, M. Becker, S. Becker, M. Beckingham, C. Becot, A. J. Beddall, A. Beddall, V. A. Bednyakov, C. P. Bee, L. J. Beemster, T. A. Beermann, M. Begel, J. K. Behr, C. Belanger-Champagne, P. J. Bell, W. H. Bell, G. Bella, L. Bellagamba, A. Bellerive, M. Bellomo, K. Belotskiy, O. Beltramello, O. Benary, D. Benchekroun, M. Bender, K. Bendtz, N. Benekos, Y. Benhammou, E. Benhar Noccioli, J. A. Benitez Garcia, D. P. Benjamin, J. R. Bensinger, S. Bentvelsen, L. Beresford, M. Beretta, D. Berge, E. Bergeaas Kuutmann, N. Berger, F. Berghaus, J. Beringer, C. Bernard, N. R. Bernard, C. Bernius, F. U. Bernlochner, T. Berry, P. Berta, C. Bertella, G. Bertoli, F. Bertolucci, C. Bertsche, D. Bertsche, M. I. Besana, G. J. Besjes, O. Bessidskaia Bylund, M. Bessner, N. Besson, C. Betancourt, S. Bethke, A. J. Bevan, W. Bhimji, R. M. Bianchi, L. Bianchini, M. Bianco, O. Biebel, S. P. Bieniek, M. Biglietti, J. Bilbao De Mendizabal, H. Bilokon, M. Bindi, S. Binet, A. Bingul, C. Bini, C. W. Black, J. E. Black, K. M. Black, D. Blackburn, R. E. Blair, J.-B. Blanchard, J. E. Blanco, T. Blazek, I. Bloch, C. Blocker, W. Blum, U. Blumenschein, G. J. Bobbink, V. S. Bobrovnikov, S. S. Bocchetta, A. Bocci, C. Bock, M. Boehler, J. A. Bogaerts, A. G. Bogdanchikov, C. Bohm, V. Boisvert, T. Bold, V. Boldea, A. S. Boldyrev, M. Bomben, M. Bona, M. Boonekamp, A. Borisov, G. Borissov, S. Borroni, J. Bortfeldt, V. Bortolotto, K. Bos, D. Boscherini, M. Bosman, J. Boudreau, J. Bouffard, E. V. Bouhova-Thacker, D. Boumediene, C. Bourdarios, N. Bousson, A. Boveia, J. Boyd, I. R. Boyko, I. Bozic, J. Bracinik, A. Brandt, G. Brandt, O. Brandt, U. Bratzler, B. Brau, J. E. Brau, H. M. Braun, S. F. Brazzale, K. Brendlinger, A. J. Brennan, L. Brenner, R. Brenner, S. Bressler, K. Bristow, T. M. Bristow, D. Britton, D. Britzger, F. M. Brochu, I. Brock, R. Brock, J. Bronner, G. Brooijmans, T. Brooks, W. K. Brooks, J. Brosamer, E. Brost, J. Brown, P. A. Bruckman de Renstrom, D. Bruncko, R. Bruneliere, A. Bruni, G. Bruni, M. Bruschi, L. Bryngemark, T. Buanes, Q. Buat, P. Buchholz, A. G. Buckley, S. I. Buda, I. A. Budagov, F. Buehrer, L. Bugge, M. K. Bugge, O. Bulekov, H. Burckhart, S. Burdin, B. Burghgrave, S. Burke, I. Burmeister, E. Busato, D. Büscher, V. Büscher, P. Bussey, C. P. Buszello, J. M. Butler, A. I. Butt, C. M. Buttar, J. M. Butterworth, P. Butti, W. Buttinger, A. Buzatu, R. Buzykaev, S. Cabrera Urbán, D. Caforio, V. M. Cairo, O. Cakir, P. Calafiura, A. Calandri, G. Calderini, P. Calfayan, L. P. Caloba, D. Calvet, S. Calvet, R. Camacho Toro, S. Camarda, P. Camarri, D. Cameron, L. M. Caminada, R. Caminal Armadans, S. Campana, M. Campanelli, A. Campoverde, V. Canale, A. Canepa, M. Cano Bret, J. Cantero, R. Cantrill, T. Cao, M. D. M. Capeans Garrido, I. Caprini, M. Caprini, M. Capua, R. Caputo, R. Cardarelli, T. Carli, G. Carlino, L. Carminati, S. Caron, E. Carquin, G. D. Carrillo-Montoya, J. R. Carter, J. Carvalho, D. Casadei, M. P. Casado, M. Casolino, E. Castaneda-Miranda, A. Castelli, V. Castillo Gimenez, N. F. Castro, P. Catastini, A. Catinaccio, J. R. Catmore, A. Cattai, J. Caudron, V. Cavaliere, D. Cavalli, M. Cavalli-Sforza, V. Cavasinni, F. Ceradini, B. C. Cerio, K. Cerny, A. S. Cerqueira, A. Cerri, L. Cerrito, F. Cerutti, M. Cerv, A. Cervelli, S. A. Cetin, A. Chafaq, D. Chakraborty, I. Chalupkova, P. Chang, B. Chapleau, J. D. Chapman, D. G. Charlton, C. C. Chau, C. A. Chavez Barajas, S. Cheatham, A. Chegwidden, S. Chekanov, S. V. Chekulaev, G. A. Chelkov, M. A. Chelstowska, C. Chen, H. Chen, K. Chen, L. Chen, S. Chen, X. Chen, Y. Chen, H. C. Cheng, Y. Cheng, A. Cheplakov, E. Cheremushkina, R. Cherkaoui El Moursli, V. Chernyatin, E. Cheu, L. Chevalier, V. Chiarella, J. T. Childers, G. Chiodini, A. S. Chisholm, R. T. Chislett, A. Chitan, M. V. Chizhov, K. Choi, S. Chouridou, B. K. B. Chow, V. Christodoulou, D. Chromek-Burckhart, M. L. Chu, J. Chudoba, A. J. Chuinard, J. J. Chwastowski, L. Chytka, G. Ciapetti, A. K. Ciftci, D. Cinca, V. Cindro, I. A. Cioara, A. Ciocio, Z. H. Citron, M. Ciubancan, A. Clark, B. L. Clark, P. J. Clark, R. N. Clarke, W. Cleland, C. Clement, Y. Coadou, M. Cobal, A. Coccaro, J. Cochran, L. Coffey, J. G. Cogan, B. Cole, S. Cole, A. P. Colijn, J. Collot, T. Colombo, G. Compostella, P. Conde Muiño, E. Coniavitis, S. H. Connell, I. A. Connelly, S. M. Consonni, V. Consorti, S. Constantinescu, C. Conta, G. Conti, F. Conventi, M. Cooke, B. D. Cooper, A. M. Cooper-Sarkar, T. Cornelissen, M. Corradi, F. Corriveau, A. Corso-Radu, A. Cortes-Gonzalez, G. Cortiana, G. Costa, M. J. Costa, D. Costanzo, D. Côté, G. Cottin, G. Cowan, B. E. Cox, K. Cranmer, G. Cree, S. Crépé-Renaudin, F. Crescioli, W. A. Cribbs, M. Crispin Ortuzar, M. Cristinziani, V. Croft, G. Crosetti, T. Cuhadar Donszelmann, J. Cummings, M. Curatolo, C. Cuthbert, H. Czirr, P. Czodrowski, S. D’Auria, M. D’Onofrio, M. J. Da Cunha Sargedas De Sousa, C. Da Via, W. Dabrowski, A. Dafinca, T. Dai, O. Dale, F. Dallaire, C. Dallapiccola, M. Dam, J. R. Dandoy, N. P. Dang, A. C. Daniells, M. Danninger, M. Dano Hoffmann, V. Dao, G. Darbo, S. Darmora, J. Dassoulas, A. Dattagupta, W. Davey, C. David, T. Davidek, E. Davies, M. Davies, P. Davison, Y. Davygora, E. Dawe, I. Dawson, R. K. Daya-Ishmukhametova, K. De, R. de Asmundis, S. De Castro, S. De Cecco, N. De Groot, P. de Jong, H. De la Torre, F. De Lorenzi, L. De Nooij, D. De Pedis, A. De Salvo, U. De Sanctis, A. De Santo, J. B. De Vivie De Regie, W. J. Dearnaley, R. Debbe, C. Debenedetti, D. V. Dedovich, I. Deigaard, J. Del Peso, T. Del Prete, D. Delgove, F. Deliot, C. M. Delitzsch, M. Deliyergiyev, A. Dell’Acqua, L. Dell’Asta, M. Dell’Orso, M. Della Pietra, D. della Volpe, M. Delmastro, P. A. Delsart, C. Deluca, D. A. DeMarco, S. Demers, M. Demichev, A. Demilly, S. P. Denisov, D. Derendarz, J. E. Derkaoui, F. Derue, P. Dervan, K. Desch, C. Deterre, P. O. Deviveiros, A. Dewhurst, S. Dhaliwal, A. Di Ciaccio, L. Di Ciaccio, A. Di Domenico, C. Di Donato, A. Di Girolamo, B. Di Girolamo, A. Di Mattia, B. Di Micco, R. Di Nardo, A. Di Simone, R. Di Sipio, D. Di Valentino, C. Diaconu, M. Diamond, F. A. Dias, M. A. Diaz, E. B. Diehl, J. Dietrich, S. Diglio, A. Dimitrievska, J. Dingfelder, F. Dittus, F. Djama, T. Djobava, J. I. Djuvsland, M. A. B. do Vale, D. Dobos, M. Dobre, C. Doglioni, T. Dohmae, J. Dolejsi, Z. Dolezal, B. A. Dolgoshein, M. Donadelli, S. Donati, P. Dondero, J. Donini, J. Dopke, A. Doria, M. T. Dova, A. T. Doyle, E. Drechsler, M. Dris, E. Dubreuil, E. Duchovni, G. Duckeck, O. A. Ducu, D. Duda, A. Dudarev, L. Duflot, L. Duguid, M. Dührssen, M. Dunford, H. Duran Yildiz, M. Düren, A. Durglishvili, D. Duschinger, M. Dyndal, C. Eckardt, K. M. Ecker, R. C. Edgar, W. Edson, N. C. Edwards, W. Ehrenfeld, T. Eifert, G. Eigen, K. Einsweiler, T. Ekelof, M. El Kacimi, M. Ellert, S. Elles, F. Ellinghaus, A. A. Elliot, N. Ellis, J. Elmsheuser, M. Elsing, D. Emeliyanov, Y. Enari, O. C. Endner, M. Endo, R. Engelmann, J. Erdmann, A. Ereditato, G. Ernis, J. Ernst, M. Ernst, S. Errede, E. Ertel, M. Escalier, H. Esch, C. Escobar, B. Esposito, A. I. Etienvre, E. Etzion, H. Evans, A. Ezhilov, L. Fabbri, G. Facini, R. M. Fakhrutdinov, S. Falciano, R. J. Falla, J. Faltova, Y. Fang, M. Fanti, A. Farbin, A. Farilla, T. Farooque, S. Farrell, S. M. Farrington, P. Farthouat, F. Fassi, P. Fassnacht, D. Fassouliotis, M. Faucci Giannelli, A. Favareto, L. Fayard, P. Federic, O. L. Fedin, W. Fedorko, S. Feigl, L. Feligioni, C. Feng, E. J. Feng, H. Feng, A. B. Fenyuk, P. Fernandez Martinez, S. Fernandez Perez, S. Ferrag, J. Ferrando, A. Ferrari, P. Ferrari, R. Ferrari, D. E. Ferreira de Lima, A. Ferrer, D. Ferrere, C. Ferretti, A. Ferretto Parodi, M. Fiascaris, F. Fiedler, A. Filipčič, M. Filipuzzi, F. Filthaut, M. Fincke-Keeler, K. D. Finelli, M. C. N. Fiolhais, L. Fiorini, A. Firan, A. Fischer, C. Fischer, J. Fischer, W. C. Fisher, E. A. Fitzgerald, M. Flechl, I. Fleck, P. Fleischmann, S. Fleischmann, G. T. Fletcher, G. Fletcher, T. Flick, A. Floderus, L. R. Flores Castillo, M. J. Flowerdew, A. Formica, A. Forti, D. Fournier, H. Fox, S. Fracchia, P. Francavilla, M. Franchini, D. Francis, L. Franconi, M. Franklin, M. Fraternali, D. Freeborn, S. T. French, F. Friedrich, D. Froidevaux, J. A. Frost, C. Fukunaga, E. Fullana Torregrosa, B. G. Fulsom, J. Fuster, C. Gabaldon, O. Gabizon, A. Gabrielli, A. Gabrielli, S. Gadatsch, S. Gadomski, G. Gagliardi, P. Gagnon, C. Galea, B. Galhardo, E. J. Gallas, B. J. Gallop, P. Gallus, G. Galster, K. K. Gan, J. Gao, Y. Gao, Y. S. Gao, F. M. Garay Walls, F. Garberson, C. García, J. E. García Navarro, M. Garcia-Sciveres, R. W. Gardner, N. Garelli, V. Garonne, C. Gatti, A. Gaudiello, G. Gaudio, B. Gaur, L. Gauthier, P. Gauzzi, I. L. Gavrilenko, C. Gay, G. Gaycken, E. N. Gazis, P. Ge, Z. Gecse, C. N. P. Gee, D. A. A. Geerts, Ch. Geich-Gimbel, M. P. Geisler, C. Gemme, M. H. Genest, S. Gentile, M. George, S. George, D. Gerbaudo, A. Gershon, H. Ghazlane, B. Giacobbe, S. Giagu, V. Giangiobbe, P. Giannetti, B. Gibbard, S. M. Gibson, M. Gilchriese, T. P. S. Gillam, D. Gillberg, G. Gilles, D. M. Gingrich, N. Giokaris, M. P. Giordani, F. M. Giorgi, F. M. Giorgi, P. F. Giraud, P. Giromini, D. Giugni, C. Giuliani, M. Giulini, B. K. Gjelsten, S. Gkaitatzis, I. Gkialas, E. L. Gkougkousis, L. K. Gladilin, C. Glasman, J. Glatzer, P. C. F. Glaysher, A. Glazov, M. Goblirsch-Kolb, J. R. Goddard, J. Godlewski, S. Goldfarb, T. Golling, D. Golubkov, A. Gomes, R. Gonçalo, J. Goncalves Pinto Firmino Da Costa, L. Gonella, S. González de la Hoz, G. Gonzalez Parra, S. Gonzalez-Sevilla, L. Goossens, P. A. Gorbounov, H. A. Gordon, I. Gorelov, B. Gorini, E. Gorini, A. Gorišek, E. Gornicki, A. T. Goshaw, C. Gössling, M. I. Gostkin, D. Goujdami, A. G. Goussiou, N. Govender, H. M. X. Grabas, L. Graber, I. Grabowska-Bold, P. Grafström, K-J. Grahn, J. Gramling, E. Gramstad, S. Grancagnolo, V. Grassi, V. Gratchev, H. M. Gray, E. Graziani, Z. D. Greenwood, K. Gregersen, I. M. Gregor, P. Grenier, J. Griffiths, A. A. Grillo, K. Grimm, S. Grinstein, Ph. Gris, J.-F. Grivaz, J. P. Grohs, A. Grohsjean, E. Gross, J. Grosse-Knetter, G. C. Grossi, Z. J. Grout, L. Guan, J. Guenther, F. Guescini, D. Guest, O. Gueta, E. Guido, T. Guillemin, S. Guindon, U. Gul, C. Gumpert, J. Guo, S. Gupta, P. Gutierrez, N. G. Gutierrez Ortiz, C. Gutschow, C. Guyot, C. Gwenlan, C. B. Gwilliam, A. Haas, C. Haber, H. K. Hadavand, N. Haddad, P. Haefner, S. Hageböck, Z. Hajduk, H. Hakobyan, M. Haleem, J. Haley, D. Hall, G. Halladjian, G. D. Hallewell, K. Hamacher, P. Hamal, K. Hamano, M. Hamer, A. Hamilton, S. Hamilton, G. N. Hamity, P. G. Hamnett, L. Han, K. Hanagaki, K. Hanawa, M. Hance, P. Hanke, R. Hanna, J. B. Hansen, J. D. Hansen, M. C. Hansen, P. H. Hansen, K. Hara, A. S. Hard, T. Harenberg, F. Hariri, S. Harkusha, R. D. Harrington, P. F. Harrison, F. Hartjes, M. Hasegawa, S. Hasegawa, Y. Hasegawa, A. Hasib, S. Hassani, S. Haug, R. Hauser, L. Hauswald, M. Havranek, C. M. Hawkes, R. J. Hawkings, A. D. Hawkins, T. Hayashi, D. Hayden, C. P. Hays, J. M. Hays, H. S. Hayward, S. J. Haywood, S. J. Head, T. Heck, V. Hedberg, L. Heelan, S. Heim, T. Heim, B. Heinemann, L. Heinrich, J. Hejbal, L. Helary, S. Hellman, D. Hellmich, C. Helsens, J. Henderson, R. C. W. Henderson, Y. Heng, C. Hengler, A. Henrichs, A. M. Henriques Correia, S. Henrot-Versille, G. H. Herbert, Y. Hernández Jiménez, R. Herrberg-Schubert, G. Herten, R. Hertenberger, L. Hervas, G. G. Hesketh, N. P. Hessey, J. W. Hetherly, R. Hickling, E. Higón-Rodriguez, E. Hill, J. C. Hill, K. H. Hiller, S. J. Hillier, I. Hinchliffe, E. Hines, R. R. Hinman, M. Hirose, D. Hirschbuehl, J. Hobbs, N. Hod, M. C. Hodgkinson, P. Hodgson, A. Hoecker, M. R. Hoeferkamp, F. Hoenig, M. Hohlfeld, D. Hohn, T. R. Holmes, T. M. Hong, L. Hooft van Huysduynen, W. H. Hopkins, Y. Horii, A. J. Horton, J-Y. Hostachy, S. Hou, A. Hoummada, J. Howard, J. Howarth, M. Hrabovsky, I. Hristova, J. Hrivnac, T. Hryn’ova, A. Hrynevich, C. Hsu, P. J. Hsu, S.-C. Hsu, D. Hu, Q. Hu, X. Hu, Y. Huang, Z. Hubacek, F. Hubaut, F. Huegging, T. B. Huffman, E. W. Hughes, G. Hughes, M. Huhtinen, T. A. Hülsing, N. Huseynov, J. Huston, J. Huth, G. Iacobucci, G. Iakovidis, I. Ibragimov, L. Iconomidou-Fayard, E. Ideal, Z. Idrissi, P. Iengo, O. Igonkina, T. Iizawa, Y. Ikegami, K. Ikematsu, M. Ikeno, Y. Ilchenko, D. Iliadis, N. Ilic, Y. Inamaru, T. Ince, P. Ioannou, M. Iodice, K. Iordanidou, V. Ippolito, A. Irles Quiles, C. Isaksson, M. Ishino, M. Ishitsuka, R. Ishmukhametov, C. Issever, S. Istin, J. M. Iturbe Ponce, R. Iuppa, J. Ivarsson, W. Iwanski, H. Iwasaki, J. M. Izen, V. Izzo, S. Jabbar, B. Jackson, M. Jackson, P. Jackson, M. R. Jaekel, V. Jain, K. Jakobs, S. Jakobsen, T. Jakoubek, J. Jakubek, D. O. Jamin, D. K. Jana, E. Jansen, R. W. Jansky, J. Janssen, M. Janus, G. Jarlskog, N. Javadov, T. Javůrek, L. Jeanty, J. Jejelava, G.-Y. Jeng, D. Jennens, P. Jenni, J. Jentzsch, C. Jeske, S. Jézéquel, H. Ji, J. Jia, Y. Jiang, S. Jiggins, J. Jimenez Pena, S. Jin, A. Jinaru, O. Jinnouchi, M. D. Joergensen, P. Johansson, K. A. Johns, K. Jon-And, G. Jones, R. W. L. Jones, T. J. Jones, J. Jongmanns, P. M. Jorge, K. D. Joshi, J. Jovicevic, X. Ju, C. A. Jung, P. Jussel, A. Juste Rozas, M. Kaci, A. Kaczmarska, M. Kado, H. Kagan, M. Kagan, S. J. Kahn, E. Kajomovitz, C. W. Kalderon, S. Kama, A. Kamenshchikov, N. Kanaya, M. Kaneda, S. Kaneti, V. A. Kantserov, J. Kanzaki, B. Kaplan, A. Kapliy, D. Kar, K. Karakostas, A. Karamaoun, N. Karastathis, M. J. Kareem, M. Karnevskiy, S. N. Karpov, Z. M. Karpova, K. Karthik, V. Kartvelishvili, A. N. Karyukhin, L. Kashif, R. D. Kass, A. Kastanas, Y. Kataoka, A. Katre, J. Katzy, K. Kawagoe, T. Kawamoto, G. Kawamura, S. Kazama, V. F. Kazanin, M. Y. Kazarinov, R. Keeler, R. Kehoe, J. S. Keller, J. J. Kempster, H. Keoshkerian, O. Kepka, B. P. Kerševan, S. Kersten, R. A. Keyes, F. Khalil-zada, H. Khandanyan, A. Khanov, A. G. Kharlamov, T. J. Khoo, V. Khovanskiy, E. Khramov, J. Khubua, H. Y. Kim, H. Kim, S. H. Kim, Y. Kim, N. Kimura, O. M. Kind, B. T. King, M. King, R. S. B. King, S. B. King, J. Kirk, A. E. Kiryunin, T. Kishimoto, D. Kisielewska, F. Kiss, K. Kiuchi, O. Kivernyk, E. Kladiva, M. H. Klein, M. Klein, U. Klein, K. Kleinknecht, P. Klimek, A. Klimentov, R. Klingenberg, J. A. Klinger, T. Klioutchnikova, P. F. Klok, E.-E. Kluge, P. Kluit, S. Kluth, E. Kneringer, E. B. F. G. Knoops, A. Knue, A. Kobayashi, D. Kobayashi, T. Kobayashi, M. Kobel, M. Kocian, P. Kodys, T. Koffas, E. Koffeman, L. A. Kogan, S. Kohlmann, Z. Kohout, T. Kohriki, T. Koi, H. Kolanoski, I. Koletsou, A. A. Komar, Y. Komori, T. Kondo, N. Kondrashova, K. Köneke, A. C. König, S. König, T. Kono, R. Konoplich, N. Konstantinidis, R. Kopeliansky, S. Koperny, L. Köpke, A. K. Kopp, K. Korcyl, K. Kordas, A. Korn, A. A. Korol, I. Korolkov, E. V. Korolkova, O. Kortner, S. Kortner, T. Kosek, V. V. Kostyukhin, V. M. Kotov, A. Kotwal, A. Kourkoumeli-Charalampidi, C. Kourkoumelis, V. Kouskoura, A. Koutsman, R. Kowalewski, T. Z. Kowalski, W. Kozanecki, A. S. Kozhin, V. A. Kramarenko, G. Kramberger, D. Krasnopevtsev, M. W. Krasny, A. Krasznahorkay, J. K. Kraus, A. Kravchenko, S. Kreiss, M. Kretz, J. Kretzschmar, K. Kreutzfeldt, P. Krieger, K. Krizka, K. Kroeninger, H. Kroha, J. Kroll, J. Kroseberg, J. Krstic, U. Kruchonak, H. Krüger, N. Krumnack, Z. V. Krumshteyn, A. Kruse, M. C. Kruse, M. Kruskal, T. Kubota, H. Kucuk, S. Kuday, S. Kuehn, A. Kugel, F. Kuger, A. Kuhl, T. Kuhl, V. Kukhtin, Y. Kulchitsky, S. Kuleshov, M. Kuna, T. Kunigo, A. Kupco, H. Kurashige, Y. A. Kurochkin, R. Kurumida, V. Kus, E. S. Kuwertz, M. Kuze, J. Kvita, T. Kwan, D. Kyriazopoulos, A. La Rosa, J. L. La Rosa Navarro, L. La Rotonda, C. Lacasta, F. Lacava, J. Lacey, H. Lacker, D. Lacour, V. R. Lacuesta, E. Ladygin, R. Lafaye, B. Laforge, T. Lagouri, S. Lai, L. Lambourne, S. Lammers, C. L. Lampen, W. Lampl, E. Lançon, U. Landgraf, M. P. J. Landon, V. S. Lang, J. C. Lange, A. J. Lankford, F. Lanni, K. Lantzsch, S. Laplace, C. Lapoire, J. F. Laporte, T. Lari, F. Lasagni Manghi, M. Lassnig, P. Laurelli, W. Lavrijsen, A. T. Law, P. Laycock, O. Le Dortz, E. Le Guirriec, E. Le Menedeu, M. LeBlanc, T. LeCompte, F. Ledroit-Guillon, C. A. Lee, S. C. Lee, L. Lee, G. Lefebvre, M. Lefebvre, F. Legger, C. Leggett, A. Lehan, G. Lehmann Miotto, X. Lei, W. A. Leight, A. Leisos, A. G. Leister, M. A. L. Leite, R. Leitner, D. Lellouch, B. Lemmer, K. J. C. Leney, T. Lenz, B. Lenzi, R. Leone, S. Leone, C. Leonidopoulos, S. Leontsinis, C. Leroy, C. G. Lester, M. Levchenko, J. Levêque, D. Levin, L. J. Levinson, M. Levy, A. Lewis, A. M. Leyko, M. Leyton, B. Li, H. Li, H. L. Li, L. Li, L. Li, S. Li, Y. Li, Z. Liang, H. Liao, B. Liberti, A. Liblong, P. Lichard, K. Lie, J. Liebal, W. Liebig, C. Limbach, A. Limosani, S. C. Lin, T. H. Lin, F. Linde, B. E. Lindquist, J. T. Linnemann, E. Lipeles, A. Lipniacka, M. Lisovyi, T. M. Liss, D. Lissauer, A. Lister, A. M. Litke, B. Liu, D. Liu, J. Liu, J. B. Liu, K. Liu, L. Liu, M. Liu, M. Liu, Y. Liu, M. Livan, A. Lleres, J. Llorente Merino, S. L. Lloyd, F. Lo Sterzo, E. Lobodzinska, P. Loch, W. S. Lockman, F. K. Loebinger, A. E. Loevschall-Jensen, A. Loginov, T. Lohse, K. Lohwasser, M. Lokajicek, B. A. Long, J. D. Long, R. E. Long, K. A. Looper, L. Lopes, D. Lopez Mateos, B. Lopez Paredes, I. Lopez Paz, J. Lorenz, N. Lorenzo Martinez, M. Losada, P. Loscutoff, P. J. Lösel, X. Lou, A. Lounis, J. Love, P. A. Love, N. Lu, H. J. Lubatti, C. Luci, A. Lucotte, F. Luehring, W. Lukas, L. Luminari, O. Lundberg, B. Lund-Jensen, D. Lynn, R. Lysak, E. Lytken, H. Ma, L. L. Ma, G. Maccarrone, A. Macchiolo, C. M. Macdonald, J. Machado Miguens, D. Macina, D. Madaffari, R. Madar, H. J. Maddocks, W. F. Mader, A. Madsen, S. Maeland, T. Maeno, A. Maevskiy, E. Magradze, K. Mahboubi, J. Mahlstedt, C. Maiani, C. Maidantchik, A. A. Maier, T. Maier, A. Maio, S. Majewski, Y. Makida, N. Makovec, B. Malaescu, Pa. Malecki, V. P. Maleev, F. Malek, U. Mallik, D. Malon, C. Malone, S. Maltezos, V. M. Malyshev, S. Malyukov, J. Mamuzic, G. Mancini, B. Mandelli, L. Mandelli, I. Mandić, R. Mandrysch, J. Maneira, A. Manfredini, L. Manhaes de Andrade Filho, J. Manjarres Ramos, A. Mann, P. M. Manning, A. Manousakis-Katsikakis, B. Mansoulie, R. Mantifel, M. Mantoani, L. Mapelli, L. March, G. Marchiori, M. Marcisovsky, C. P. Marino, M. Marjanovic, F. Marroquim, S. P. Marsden, Z. Marshall, L. F. Marti, S. Marti-Garcia, B. Martin, T. A. Martin, V. J. Martin, B. Martin dit Latour, M. Martinez, S. Martin-Haugh, V. S. Martoiu, A. C. Martyniuk, M. Marx, F. Marzano, A. Marzin, L. Masetti, T. Mashimo, R. Mashinistov, J. Masik, A. L. Maslennikov, I. Massa, L. Massa, N. Massol, P. Mastrandrea, A. Mastroberardino, T. Masubuchi, P. Mättig, J. Mattmann, J. Maurer, S. J. Maxfield, D. A. Maximov, R. Mazini, S. M. Mazza, L. Mazzaferro, G. Mc Goldrick, S. P. Mc Kee, A. McCarn, R. L. McCarthy, T. G. McCarthy, N. A. McCubbin, K. W. McFarlane, J. A. Mcfayden, G. Mchedlidze, S. J. McMahon, R. A. McPherson, M. Medinnis, S. Meehan, S. Mehlhase, A. Mehta, K. Meier, C. Meineck, B. Meirose, B. R. Mellado Garcia, F. Meloni, A. Mengarelli, S. Menke, E. Meoni, K. M. Mercurio, S. Mergelmeyer, P. Mermod, L. Merola, C. Meroni, F. S. Merritt, A. Messina, J. Metcalfe, A. S. Mete, C. Meyer, C. Meyer, J-P. Meyer, J. Meyer, R. P. Middleton, S. Miglioranzi, L. Mijović, G. Mikenberg, M. Mikestikova, A. Mikuž, A. Milesi, A. Milic, D. W. Miller, C. Mills, A. Milov, D. A. Milstead, A. A. Minaenko, Y. Minami, I. A. Minashvili, A. I. Mincer, B. Mindur, M. Mineev, Y. Ming, L. M. Mir, T. Mitani, J. Mitrevski, V. A. Mitsou, A. Miucci, P. S. Miyagawa, J. U. Mjörnmark, T. Moa, K. Mochizuki, S. Mohapatra, W. Mohr, S. Molander, R. Moles-Valls, K. Mönig, C. Monini, J. Monk, E. Monnier, J. Montejo Berlingen, F. Monticelli, S. Monzani, R. W. Moore, N. Morange, D. Moreno, M. Moreno Llácer, P. Morettini, M. Morgenstern, M. Morii, M. Morinaga, V. Morisbak, S. Moritz, A. K. Morley, G. Mornacchi, J. D. Morris, S. S. Mortensen, A. Morton, L. Morvaj, H. G. Moser, M. Mosidze, J. Moss, K. Motohashi, R. Mount, E. Mountricha, S. V. Mouraviev, E. J. W. Moyse, S. Muanza, R. D. Mudd, F. Mueller, J. Mueller, K. Mueller, R. S. P. Mueller, T. Mueller, D. Muenstermann, P. Mullen, Y. Munwes, J. A. Murillo Quijada, W. J. Murray, H. Musheghyan, E. Musto, A. G. Myagkov, M. Myska, O. Nackenhorst, J. Nadal, K. Nagai, R. Nagai, Y. Nagai, K. Nagano, A. Nagarkar, Y. Nagasaka, K. Nagata, M. Nagel, E. Nagy, A. M. Nairz, Y. Nakahama, K. Nakamura, T. Nakamura, I. Nakano, H. Namasivayam, R. F. Naranjo Garcia, R. Narayan, T. Naumann, G. Navarro, R. Nayyar, H. A. Neal, P. Yu. Nechaeva, T. J. Neep, P. D. Nef, A. Negri, M. Negrini, S. Nektarijevic, C. Nellist, A. Nelson, S. Nemecek, P. Nemethy, A. A. Nepomuceno, M. Nessi, M. S. Neubauer, M. Neumann, R. M. Neves, P. Nevski, P. R. Newman, D. H. Nguyen, R. B. Nickerson, R. Nicolaidou, B. Nicquevert, J. Nielsen, N. Nikiforou, A. Nikiforov, V. Nikolaenko, I. Nikolic-Audit, K. Nikolopoulos, J. K. Nilsen, P. Nilsson, Y. Ninomiya, A. Nisati, R. Nisius, T. Nobe, M. Nomachi, I. Nomidis, T. Nooney, S. Norberg, M. Nordberg, O. Novgorodova, S. Nowak, M. Nozaki, L. Nozka, K. Ntekas, G. Nunes Hanninger, T. Nunnemann, E. Nurse, F. Nuti, B. J. O’Brien, F. O’grady, D. C. O’Neil, V. O’Shea, F. G. Oakham, H. Oberlack, T. Obermann, J. Ocariz, A. Ochi, I. Ochoa, S. Oda, S. Odaka, H. Ogren, A. Oh, S. H. Oh, C. C. Ohm, H. Ohman, H. Oide, W. Okamura, H. Okawa, Y. Okumura, T. Okuyama, A. Olariu, S. A. Olivares Pino, D. Oliveira Damazio, E. Oliver Garcia, A. Olszewski, J. Olszowska, A. Onofre, P. U. E. Onyisi, C. J. Oram, M. J. Oreglia, Y. Oren, D. Orestano, N. Orlando, C. Oropeza Barrera, R. S. Orr, B. Osculati, R. Ospanov, G. Otero y Garzon, H. Otono, M. Ouchrif, E. A. Ouellette, F. Ould-Saada, A. Ouraou, K. P. Oussoren, Q. Ouyang, A. Ovcharova, M. Owen, R. E. Owen, V. E. Ozcan, N. Ozturk, K. Pachal, A. Pacheco Pages, C. Padilla Aranda, M. Pagáčová, S. Pagan Griso, E. Paganis, C. Pahl, F. Paige, P. Pais, K. Pajchel, G. Palacino, S. Palestini, M. Palka, D. Pallin, A. Palma, Y. B. Pan, E. Panagiotopoulou, C. E. Pandini, J. G. Panduro Vazquez, P. Pani, S. Panitkin, L. Paolozzi, Th. D. Papadopoulou, K. Papageorgiou, A. Paramonov, D. Paredes Hernandez, M. A. Parker, K. A. Parker, F. Parodi, J. A. Parsons, U. Parzefall, E. Pasqualucci, S. Passaggio, F. Pastore, Fr. Pastore, G. Pásztor, S. Pataraia, N. D. Patel, J. R. Pater, T. Pauly, J. Pearce, B. Pearson, L. E. Pedersen, M. Pedersen, S. Pedraza Lopez, R. Pedro, S. V. Peleganchuk, D. Pelikan, H. Peng, B. Penning, J. Penwell, D. V. Perepelitsa, E. Perez Codina, M. T. Pérez García-Estañ, L. Perini, H. Pernegger, S. Perrella, R. Peschke, V. D. Peshekhonov, K. Peters, R. F. Y. Peters, B. A. Petersen, T. C. Petersen, E. Petit, A. Petridis, C. Petridou, E. Petrolo, F. Petrucci, N. E. Pettersson, R. Pezoa, P. W. Phillips, G. Piacquadio, E. Pianori, A. Picazio, E. Piccaro, M. Piccinini, M. A. Pickering, R. Piegaia, D. T. Pignotti, J. E. Pilcher, A. D. Pilkington, J. Pina, M. Pinamonti, J. L. Pinfold, A. Pingel, B. Pinto, S. Pires, M. Pitt, C. Pizio, L. Plazak, M.-A. Pleier, V. Pleskot, E. Plotnikova, P. Plucinski, D. Pluth, R. Poettgen, L. Poggioli, D. Pohl, G. Polesello, A. Policicchio, R. Polifka, A. Polini, C. S. Pollard, V. Polychronakos, K. Pommès, L. Pontecorvo, B. G. Pope, G. A. Popeneciu, D. S. Popovic, A. Poppleton, S. Pospisil, K. Potamianos, I. N. Potrap, C. J. Potter, C. T. Potter, G. Poulard, J. Poveda, V. Pozdnyakov, P. Pralavorio, A. Pranko, S. Prasad, S. Prell, D. Price, L. E. Price, M. Primavera, S. Prince, M. Proissl, K. Prokofiev, F. Prokoshin, E. Protopapadaki, S. Protopopescu, J. Proudfoot, M. Przybycien, E. Ptacek, D. Puddu, E. Pueschel, D. Puldon, M. Purohit, P. Puzo, J. Qian, G. Qin, Y. Qin, A. Quadt, D. R. Quarrie, W. B. Quayle, M. Queitsch-Maitland, D. Quilty, S. Raddum, V. Radeka, V. Radescu, S. K. Radhakrishnan, P. Radloff, P. Rados, F. Ragusa, G. Rahal, S. Rajagopalan, M. Rammensee, C. Rangel-Smith, F. Rauscher, S. Rave, T. Ravenscroft, M. Raymond, A. L. Read, N. P. Readioff, D. M. Rebuzzi, A. Redelbach, G. Redlinger, R. Reece, K. Reeves, L. Rehnisch, H. Reisin, M. Relich, C. Rembser, H. Ren, A. Renaud, M. Rescigno, S. Resconi, O. L. Rezanova, P. Reznicek, R. Rezvani, R. Richter, S. Richter, E. Richter-Was, O. Ricken, M. Ridel, P. Rieck, C. J. Riegel, J. Rieger, M. Rijssenbeek, A. Rimoldi, L. Rinaldi, B. Ristić, E. Ritsch, I. Riu, F. Rizatdinova, E. Rizvi, S. H. Robertson, A. Robichaud-Veronneau, D. Robinson, J. E. M. Robinson, A. Robson, C. Roda, S. Roe, O. Røhne, S. Rolli, A. Romaniouk, M. Romano, S. M. Romano Saez, E. Romero Adam, N. Rompotis, M. Ronzani, L. Roos, E. Ros, S. Rosati, K. Rosbach, P. Rose, P. L. Rosendahl, O. Rosenthal, V. Rossetti, E. Rossi, L. P. Rossi, R. Rosten, M. Rotaru, I. Roth, J. Rothberg, D. Rousseau, C. R. Royon, A. Rozanov, Y. Rozen, X. Ruan, F. Rubbo, I. Rubinskiy, V. I. Rud, C. Rudolph, M. S. Rudolph, F. Rühr, A. Ruiz-Martinez, Z. Rurikova, N. A. Rusakovich, A. Ruschke, H. L. Russell, J. P. Rutherfoord, N. Ruthmann, Y. F. Ryabov, M. Rybar, G. Rybkin, N. C. Ryder, A. F. Saavedra, G. Sabato, S. Sacerdoti, A. Saddique, H. F-W. Sadrozinski, R. Sadykov, F. Safai Tehrani, M. Saimpert, H. Sakamoto, Y. Sakurai, G. Salamanna, A. Salamon, M. Saleem, D. Salek, P. H. Sales De Bruin, D. Salihagic, A. Salnikov, J. Salt, D. Salvatore, F. Salvatore, A. Salvucci, A. Salzburger, D. Sampsonidis, A. Sanchez, J. Sánchez, V. Sanchez Martinez, H. Sandaker, R. L. Sandbach, H. G. Sander, M. P. Sanders, M. Sandhoff, C. Sandoval, R. Sandstroem, D. P. C. Sankey, M. Sannino, A. Sansoni, C. Santoni, R. Santonico, H. Santos, I. Santoyo Castillo, K. Sapp, A. Sapronov, J. G. Saraiva, B. Sarrazin, O. Sasaki, Y. Sasaki, K. Sato, G. Sauvage, E. Sauvan, G. Savage, P. Savard, C. Sawyer, L. Sawyer, J. Saxon, C. Sbarra, A. Sbrizzi, T. Scanlon, D. A. Scannicchio, M. Scarcella, V. Scarfone, J. Schaarschmidt, P. Schacht, D. Schaefer, R. Schaefer, J. Schaeffer, S. Schaepe, S. Schaetzel, U. Schäfer, A. C. Schaffer, D. Schaile, R. D. Schamberger, V. Scharf, V. A. Schegelsky, D. Scheirich, M. Schernau, C. Schiavi, C. Schillo, M. Schioppa, S. Schlenker, E. Schmidt, K. Schmieden, C. Schmitt, S. Schmitt, S. Schmitt, B. Schneider, Y. J. Schnellbach, U. Schnoor, L. Schoeffel, A. Schoening, B. D. Schoenrock, E. Schopf, A. L. S. Schorlemmer, M. Schott, D. Schouten, J. Schovancova, S. Schramm, M. Schreyer, C. Schroeder, N. Schuh, M. J. Schultens, H.-C. Schultz-Coulon, H. Schulz, M. Schumacher, B. A. Schumm, Ph. Schune, C. Schwanenberger, A. Schwartzman, T. A. Schwarz, Ph. Schwegler, Ph. Schwemling, R. Schwienhorst, J. Schwindling, T. Schwindt, M. Schwoerer, F. G. Sciacca, E. Scifo, G. Sciolla, F. Scuri, F. Scutti, J. Searcy, G. Sedov, E. Sedykh, P. Seema, S. C. Seidel, A. Seiden, F. Seifert, J. M. Seixas, G. Sekhniaidze, K. Sekhon, S. J. Sekula, K. E. Selbach, D. M. Seliverstov, N. Semprini-Cesari, C. Serfon, L. Serin, L. Serkin, T. Serre, M. Sessa, R. Seuster, H. Severini, T. Sfiligoj, F. Sforza, A. Sfyrla, E. Shabalina, M. Shamim, L. Y. Shan, R. Shang, J. T. Shank, M. Shapiro, P. B. Shatalov, K. Shaw, S. M. Shaw, A. Shcherbakova, C. Y. Shehu, P. Sherwood, L. Shi, S. Shimizu, C. O. Shimmin, M. Shimojima, M. Shiyakova, A. Shmeleva, D. Shoaleh Saadi, M. J. Shochet, S. Shojaii, S. Shrestha, E. Shulga, M. A. Shupe, S. Shushkevich, P. Sicho, O. Sidiropoulou, D. Sidorov, A. Sidoti, F. Siegert, Dj. Sijacki, J. Silva, Y. Silver, S. B. Silverstein, V. Simak, O. Simard, Lj. Simic, S. Simion, E. Simioni, B. Simmons, D. Simon, R. Simoniello, P. Sinervo, N. B. Sinev, G. Siragusa, A. N. Sisakyan, S. Yu. Sivoklokov, J. Sjölin, T. B. Sjursen, M. B. Skinner, H. P. Skottowe, P. Skubic, M. Slater, T. Slavicek, M. Slawinska, K. Sliwa, V. Smakhtin, B. H. Smart, L. Smestad, S. Yu. Smirnov, Y. Smirnov, L. N. Smirnova, O. Smirnova, M. N. K. Smith, M. Smizanska, K. Smolek, A. A. Snesarev, G. Snidero, S. Snyder, R. Sobie, F. Socher, A. Soffer, D. A. Soh, C. A. Solans, M. Solar, J. Solc, E. Yu. Soldatov, U. Soldevila, A. A. Solodkov, A. Soloshenko, O. V. Solovyanov, V. Solovyev, P. Sommer, H. Y. Song, N. Soni, A. Sood, A. Sopczak, B. Sopko, V. Sopko, V. Sorin, D. Sosa, M. Sosebee, C. L. Sotiropoulou, R. Soualah, P. Soueid, A. M. Soukharev, D. South, S. Spagnolo, M. Spalla, F. Spanò, W. R. Spearman, F. Spettel, R. Spighi, G. Spigo, L. A. Spiller, M. Spousta, T. Spreitzer, R. D. St. Denis, S. Staerz, J. Stahlman, R. Stamen, S. Stamm, E. Stanecka, C. Stanescu, M. Stanescu-Bellu, M. M. Stanitzki, S. Stapnes, E. A. Starchenko, J. Stark, P. Staroba, P. Starovoitov, R. Staszewski, P. Stavina, P. Steinberg, B. Stelzer, H. J. Stelzer, O. Stelzer-Chilton, H. Stenzel, S. Stern, G. A. Stewart, J. A. Stillings, M. C. Stockton, M. Stoebe, G. Stoicea, P. Stolte, S. Stonjek, A. R. Stradling, A. Straessner, M. E. Stramaglia, J. Strandberg, S. Strandberg, A. Strandlie, E. Strauss, M. Strauss, P. Strizenec, R. Ströhmer, D. M. Strom, R. Stroynowski, A. Strubig, S. A. Stucci, B. Stugu, N. A. Styles, D. Su, J. Su, R. Subramaniam, A. Succurro, Y. Sugaya, C. Suhr, M. Suk, V. V. Sulin, S. Sultansoy, T. Sumida, S. Sun, X. Sun, J. E. Sundermann, K. Suruliz, G. Susinno, M. R. Sutton, S. Suzuki, Y. Suzuki, M. Svatos, S. Swedish, M. Swiatlowski, I. Sykora, T. Sykora, D. Ta, C. Taccini, K. Tackmann, J. Taenzer, A. Taffard, R. Tafirout, N. Taiblum, H. Takai, R. Takashima, H. Takeda, T. Takeshita, Y. Takubo, M. Talby, A. A. Talyshev, J. Y. C. Tam, K. G. Tan, J. Tanaka, R. Tanaka, S. Tanaka, S. Tanaka, B. B. Tannenwald, N. Tannoury, S. Tapprogge, S. Tarem, F. Tarrade, G. F. Tartarelli, P. Tas, M. Tasevsky, T. Tashiro, E. Tassi, A. Tavares Delgado, Y. Tayalati, F. E. Taylor, G. N. Taylor, W. Taylor, F. A. Teischinger, M. Teixeira Dias Castanheira, P. Teixeira-Dias, K. K. Temming, H. Ten Kate, P. K. Teng, J. J. Teoh, F. Tepel, S. Terada, K. Terashi, J. Terron, S. Terzo, M. Testa, R. J. Teuscher, J. Therhaag, T. Theveneaux-Pelzer, J. P. Thomas, J. Thomas-Wilsker, E. N. Thompson, P. D. Thompson, R. J. Thompson, A. S. Thompson, L. A. Thomsen, E. Thomson, M. Thomson, R. P. Thun, M. J. Tibbetts, R. E. Ticse Torres, V. O. Tikhomirov, Yu. A. Tikhonov, S. Timoshenko, E. Tiouchichine, P. Tipton, S. Tisserant, T. Todorov, S. Todorova-Nova, J. Tojo, S. Tokár, K. Tokushuku, K. Tollefson, E. Tolley, L. Tomlinson, M. Tomoto, L. Tompkins, K. Toms, E. Torrence, H. Torres, E. Torró Pastor, J. Toth, F. Touchard, D. R. Tovey, T. Trefzger, L. Tremblet, A. Tricoli, I. M. Trigger, S. Trincaz-Duvoid, M. F. Tripiana, W. Trischuk, B. Trocmé, C. Troncon, M. Trottier-McDonald, M. Trovatelli, P. True, L. Truong, M. Trzebinski, A. Trzupek, C. Tsarouchas, J. C-L. Tseng, P. V. Tsiareshka, D. Tsionou, G. Tsipolitis, N. Tsirintanis, S. Tsiskaridze, V. Tsiskaridze, E. G. Tskhadadze, I. I. Tsukerman, V. Tsulaia, S. Tsuno, D. Tsybychev, A. Tudorache, V. Tudorache, A. N. Tuna, S. A. Tupputi, S. Turchikhin, D. Turecek, R. Turra, A. J. Turvey, P. M. Tuts, A. Tykhonov, M. Tylmad, M. Tyndel, I. Ueda, R. Ueno, M. Ughetto, M. Ugland, M. Uhlenbrock, F. Ukegawa, G. Unal, A. Undrus, G. Unel, F. C. Ungaro, Y. Unno, C. Unverdorben, J. Urban, P. Urquijo, P. Urrejola, G. Usai, A. Usanova, L. Vacavant, V. Vacek, B. Vachon, C. Valderanis, N. Valencic, S. Valentinetti, A. Valero, L. Valery, S. Valkar, E. Valladolid Gallego, S. Vallecorsa, J. A. Valls Ferrer, W. Van Den Wollenberg, P. C. Van Der Deijl, R. van der Geer, H. van der Graaf, R. Van Der Leeuw, N. van Eldik, P. van Gemmeren, J. Van Nieuwkoop, I. van Vulpen, M. C. van Woerden, M. Vanadia, W. Vandelli, R. Vanguri, A. Vaniachine, F. Vannucci, G. Vardanyan, R. Vari, E. W. Varnes, T. Varol, D. Varouchas, A. Vartapetian, K. E. Varvell, F. Vazeille, T. Vazquez Schroeder, J. Veatch, F. Veloso, T. Velz, S. Veneziano, A. Ventura, D. Ventura, M. Venturi, N. Venturi, A. Venturini, V. Vercesi, M. Verducci, W. Verkerke, J. C. Vermeulen, A. Vest, M. C. Vetterli, O. Viazlo, I. Vichou, T. Vickey, O. E. Vickey Boeriu, G. H. A. Viehhauser, S. Viel, R. Vigne, M. Villa, M. Villaplana Perez, E. Vilucchi, M. G. Vincter, V. B. Vinogradov, I. Vivarelli, F. Vives Vaque, S. Vlachos, D. Vladoiu, M. Vlasak, M. Vogel, P. Vokac, G. Volpi, M. Volpi, H. von der Schmitt, H. von Radziewski, E. von Toerne, V. Vorobel, K. Vorobev, M. Vos, R. Voss, J. H. Vossebeld, N. Vranjes, M. Vranjes Milosavljevic, V. Vrba, M. Vreeswijk, R. Vuillermet, I. Vukotic, Z. Vykydal, P. Wagner, W. Wagner, H. Wahlberg, S. Wahrmund, J. Wakabayashi, J. Walder, R. Walker, W. Walkowiak, C. Wang, F. Wang, H. Wang, H. Wang, J. Wang, J. Wang, K. Wang, R. Wang, S. M. Wang, T. Wang, X. Wang, C. Wanotayaroj, A. Warburton, C. P. Ward, D. R. Wardrope, M. Warsinsky, A. Washbrook, C. Wasicki, P. M. Watkins, A. T. Watson, I. J. Watson, M. F. Watson, G. Watts, S. Watts, B. M. Waugh, S. Webb, M. S. Weber, S. W. Weber, J. S. Webster, A. R. Weidberg, B. Weinert, J. Weingarten, C. Weiser, H. Weits, P. S. Wells, T. Wenaus, T. Wengler, S. Wenig, N. Wermes, M. Werner, P. Werner, M. Wessels, J. Wetter, K. Whalen, A. M. Wharton, A. White, M. J. White, R. White, S. White, D. Whiteson, F. J. Wickens, W. Wiedenmann, M. Wielers, P. Wienemann, C. Wiglesworth, L. A. M. Wiik-Fuchs, A. Wildauer, H. G. Wilkens, H. H. Williams, S. Williams, C. Willis, S. Willocq, A. Wilson, J. A. Wilson, I. Wingerter-Seez, F. Winklmeier, B. T. Winter, M. Wittgen, J. Wittkowski, S. J. Wollstadt, M. W. Wolter, H. Wolters, B. K. Wosiek, J. Wotschack, M. J. Woudstra, K. W. Wozniak, M. Wu, M. Wu, S. L. Wu, X. Wu, Y. Wu, T. R. Wyatt, B. M. Wynne, S. Xella, D. Xu, L. Xu, B. Yabsley, S. Yacoob, R. Yakabe, M. Yamada, Y. Yamaguchi, A. Yamamoto, S. Yamamoto, T. Yamanaka, K. Yamauchi, Y. Yamazaki, Z. Yan, H. Yang, H. Yang, Y. Yang, L. Yao, W-M. Yao, Y. Yasu, E. Yatsenko, K. H. Yau Wong, J. Ye, S. Ye, I. Yeletskikh, A. L. Yen, E. Yildirim, K. Yorita, R. Yoshida, K. Yoshihara, C. Young, C. J. S. Young, S. Youssef, D. R. Yu, J. Yu, J. M. Yu, J. Yu, L. Yuan, A. Yurkewicz, I. Yusuff, B. Zabinski, R. Zaidan, A. M. Zaitsev, J. Zalieckas, A. Zaman, S. Zambito, L. Zanello, D. Zanzi, C. Zeitnitz, M. Zeman, A. Zemla, K. Zengel, O. Zenin, T. Ženiš, D. Zerwas, D. Zhang, F. Zhang, J. Zhang, L. Zhang, R. Zhang, X. Zhang, Z. Zhang, X. Zhao, Y. Zhao, Z. Zhao, A. Zhemchugov, J. Zhong, B. Zhou, C. Zhou, L. Zhou, L. Zhou, N. Zhou, C. G. Zhu, H. Zhu, J. Zhu, Y. Zhu, X. Zhuang, K. Zhukov, A. Zibell, D. Zieminska, N. I. Zimine, C. Zimmermann, S. Zimmermann, Z. Zinonos, M. Zinser, M. Ziolkowski, L. Živković, G. Zobernig, A. Zoccoli, M. zur Nedden, G. Zurzolo, L. Zwalinski

**Affiliations:** Department of Physics, University of Adelaide, Adelaide, Australia; Physics Department, SUNY Albany, Albany, NY USA; Department of Physics, University of Alberta, Edmonton, AB Canada; Department of Physics, Ankara University, Ankara, Turkey; Istanbul Aydin University, Istanbul, Turkey; Division of Physics, TOBB University of Economics and Technology, Ankara, Turkey; LAPP, CNRS/IN2P3 and Université Savoie Mont Blanc, Annecy-le-Vieux, France; High Energy Physics Division, Argonne National Laboratory, Argonne, IL USA; Department of Physics, University of Arizona, Tucson, AZ USA; Department of Physics, The University of Texas at Arlington, Arlington, TX USA; Physics Department, University of Athens, Athens, Greece; Physics Department, National Technical University of Athens, Zografou, Greece; Institute of Physics, Azerbaijan Academy of Sciences, Baku, Azerbaijan; Institut de Física d’Altes Energies and Departament de Física de la Universitat Autònoma de Barcelona, Barcelona, Spain; Institute of Physics, University of Belgrade, Belgrade, Serbia; Department for Physics and Technology, University of Bergen, Bergen, Norway; Physics Division, Lawrence Berkeley National Laboratory and University of California, Berkeley, CA USA; Department of Physics, Humboldt University, Berlin, Germany; Albert Einstein Center for Fundamental Physics and Laboratory for High Energy Physics, University of Bern, Bern, Switzerland; School of Physics and Astronomy, University of Birmingham, Birmingham, UK; Department of Physics, Bogazici University, Istanbul, Turkey; Department of Physics, Dogus University, Istanbul, Turkey; Department of Physics Engineering, Gaziantep University, Gaziantep, Turkey; INFN Sezione di Bologna, Bologna, Italy; Dipartimento di Fisica e Astronomia, Università di Bologna, Bologna, Italy; Physikalisches Institut, University of Bonn, Bonn, Germany; Department of Physics, Boston University, Boston, MA USA; Department of Physics, Brandeis University, Waltham, MA USA; Universidade Federal do Rio De Janeiro COPPE/EE/IF, Rio de Janeiro, Brazil; Electrical Circuits Department, Federal University of Juiz de Fora (UFJF), Juiz de Fora, Brazil; Federal University of Sao Joao del Rei (UFSJ), Sao Joao del Rei, Brazil; Instituto de Fisica, Universidade de Sao Paulo, São Paulo, Brazil; Physics Department, Brookhaven National Laboratory, Upton, NY USA; National Institute of Physics and Nuclear Engineering, Bucharest, Romania; Physics Department, National Institute for Research and Development of Isotopic and Molecular Technologies, Cluj Napoca, Romania; University Politehnica Bucharest, Bucharest, Romania; West University in Timisoara, Timisoara, Romania; Departamento de Física, Universidad de Buenos Aires, Buenos Aires, Argentina; Cavendish Laboratory, University of Cambridge, Cambridge, UK; Department of Physics, Carleton University, Ottawa, ON Canada; CERN, Geneva, Switzerland; Enrico Fermi Institute, University of Chicago, Chicago, IL USA; Departamento de Física, Pontificia Universidad Católica de Chile, Santiago, Chile; Departamento de Física, Universidad Técnica Federico Santa María, Valparaiso, Chile; Institute of High Energy Physics, Chinese Academy of Sciences, Beijing, China; Department of Modern Physics, University of Science and Technology of China, Anhui, China; Department of Physics, Nanjing University, Jiangsu, China; School of Physics, Shandong University, Shandong, China; Department of Physics and Astronomy, Shanghai Key Laboratory for Particle Physics and Cosmology, Shanghai Jiao Tong University, Shanghai, China; Physics Department, Tsinghua University, 100084 Beijing, China; Laboratoire de Physique Corpusculaire, Clermont Université and Université Blaise Pascal and CNRS/IN2P3, Clermont-Ferrand, France; Nevis Laboratory, Columbia University, Irvington, NY USA; Niels Bohr Institute, University of Copenhagen, Copenhagen, Denmark; INFN Gruppo Collegato di Cosenza, Laboratori Nazionali di Frascati, Frascati, Italy; Dipartimento di Fisica, Università della Calabria, Rende, Italy; AGH University of Science and Technology, Faculty of Physics and Applied Computer Science, Krakow, Poland, Marian Smoluchowski Institute of Physics, Jagiellonian University, Kraków, Poland; Institute of Nuclear Physics, Polish Academy of Sciences, Kraków, Poland; Physics Department, Southern Methodist University, Dallas, TX USA; Physics Department, University of Texas at Dallas, Richardson, TX USA; DESY, Hamburg and Zeuthen, Germany; Institut für Experimentelle Physik IV, Technische Universität Dortmund, Dortmund, Germany; Institut für Kern- und Teilchenphysik, Technische Universität Dresden, Dresden, Germany; Department of Physics, Duke University, Durham, NC USA; SUPA-School of Physics and Astronomy, University of Edinburgh, Edinburgh, UK; INFN Laboratori Nazionali di Frascati, Frascati, Italy; Fakultät für Mathematik und Physik, Albert-Ludwigs-Universität, Freiburg, Germany; Section de Physique, Université de Genève, Geneva, Switzerland; INFN Sezione di Genova, Genova, Italy; Dipartimento di Fisica, Università di Genova, Genova, Italy; E. Andronikashvili Institute of Physics, Iv. Javakhishvili Tbilisi State University, Tbilisi, Georgia; High Energy Physics Institute, Tbilisi State University, Tbilisi, Georgia; II Physikalisches Institut, Justus-Liebig-Universität Giessen, Giessen, Germany; SUPA-School of Physics and Astronomy, University of Glasgow, Glasgow, UK; II Physikalisches Institut, Georg-August-Universität, Göttingen, Germany; Laboratoire de Physique Subatomique et de Cosmologie, Université Grenoble-Alpes, CNRS/IN2P3, Grenoble, France; Department of Physics, Hampton University, Hampton, VA USA; Laboratory for Particle Physics and Cosmology, Harvard University, Cambridge, MA USA; Kirchhoff-Institut für Physik, Ruprecht-Karls-Universität Heidelberg, Heidelberg, Germany; Physikalisches Institut, Ruprecht-Karls-Universität Heidelberg, Heidelberg, Germany; ZITI Institut für technische Informatik, Ruprecht-Karls-Universität Heidelberg, Mannheim, Germany; Faculty of Applied Information Science, Hiroshima Institute of Technology, Hiroshima, Japan; Department of Physics, The Chinese University of Hong Kong, Shatin, NT, Hong Kong; Department of Physics, The University of Hong Kong, Pok Fu Lam, Hong Kong; Department of Physics, The Hong Kong University of Science and Technology, Clear Water Bay, Kowloon, Hong Kong, China; Department of Physics, Indiana University, Bloomington, IN USA; Institut für Astro- und Teilchenphysik, Leopold-Franzens-Universität, Innsbruck, Austria; University of Iowa, Iowa City, IA USA; Department of Physics and Astronomy, Iowa State University, Ames, IA USA; Joint Institute for Nuclear Research, JINR Dubna, Dubna, Russia; KEK, High Energy Accelerator Research Organization, Tsukuba, Japan; Graduate School of Science, Kobe University, Kobe, Japan; Faculty of Science, Kyoto University, Kyoto, Japan; Kyoto University of Education, Kyoto, Japan; Department of Physics, Kyushu University, Fukuoka, Japan; Instituto de Física La Plata, Universidad Nacional de La Plata and CONICET, La Plata, Argentina; Physics Department, Lancaster University, Lancaster, UK; INFN Sezione di Lecce, Lecce, Italy; Dipartimento di Matematica e Fisica, Università del Salento, Lecce, Italy; Oliver Lodge Laboratory, University of Liverpool, Liverpool, UK; Department of Physics, Jožef Stefan Institute and University of Ljubljana, Ljubljana, Slovenia; School of Physics and Astronomy, Queen Mary University of London, London, UK; Department of Physics, Royal Holloway University of London, Surrey, UK; Department of Physics and Astronomy, University College London, London, UK; Louisiana Tech University, Ruston, LA USA; Laboratoire de Physique Nucléaire et de Hautes Energies, UPMC and Université Paris-Diderot and CNRS/IN2P3, Paris, France; Fysiska institutionen, Lunds universitet, Lund, Sweden; Departamento de Fisica Teorica C-15, Universidad Autonoma de Madrid, Madrid, Spain; Institut für Physik, Universität Mainz, Mainz, Germany; School of Physics and Astronomy, University of Manchester, Manchester, UK; CPPM, Aix-Marseille Université and CNRS/IN2P3, Marseille, France; Department of Physics, University of Massachusetts, Amherst, MA USA; Department of Physics, McGill University, Montreal, QC Canada; School of Physics, University of Melbourne, Melbourne, VIC Australia; Department of Physics, The University of Michigan, Ann Arbor, MI USA; Department of Physics and Astronomy, Michigan State University, East Lansing, MI USA; INFN Sezione di Milano, Milan, Italy; Dipartimento di Fisica, Università di Milano, Milan, Italy; B.I. Stepanov Institute of Physics, National Academy of Sciences of Belarus, Minsk, Republic of Belarus; National Scientific and Educational Centre for Particle and High Energy Physics, Minsk, Republic of Belarus; Department of Physics, Massachusetts Institute of Technology, Cambridge, MA USA; Group of Particle Physics, University of Montreal, Montreal, QC Canada; P.N. Lebedev Institute of Physics, Academy of Sciences, Moscow, Russia; Institute for Theoretical and Experimental Physics (ITEP), Moscow, Russia; National Research Nuclear University MEPhI, Moscow, Russia; D.V. Skobeltsyn Institute of Nuclear Physics, M.V. Lomonosov Moscow State University, Moscow, Russia; Fakultät für Physik, Ludwig-Maximilians-Universität München, Munich, Germany; Max-Planck-Institut für Physik (Werner-Heisenberg-Institut), Munich, Germany; Nagasaki Institute of Applied Science, Nagasaki, Japan; Graduate School of Science and Kobayashi-Maskawa Institute, Nagoya University, Nagoya, Japan; INFN Sezione di Napoli, Naples, Italy; Dipartimento di Fisica, Università di Napoli, Naples, Italy; Department of Physics and Astronomy, University of New Mexico, Albuquerque, NM USA; Institute for Mathematics, Astrophysics and Particle Physics, Radboud University Nijmegen/Nikhef, Nijmegen, The Netherlands; Nikhef National Institute for Subatomic Physics and University of Amsterdam, Amsterdam, The Netherlands; Department of Physics, Northern Illinois University, De Kalb, IL USA; Budker Institute of Nuclear Physics, SB RAS, Novosibirsk, Russia; Department of Physics, New York University, New York, NY USA; Ohio State University, Columbus, OH USA; Faculty of Science, Okayama University, Okayama, Japan; Homer L. Dodge Department of Physics and Astronomy, University of Oklahoma, Norman, OK USA; Department of Physics, Oklahoma State University, Stillwater, OK USA; Palacký University, RCPTM, Olomouc, Czech Republic; Center for High Energy Physics, University of Oregon, Eugene, OR USA; LAL, Université Paris-Sud and CNRS/IN2P3, Orsay, France; Graduate School of Science, Osaka University, Osaka, Japan; Department of Physics, University of Oslo, Oslo, Norway; Department of Physics, Oxford University, Oxford, UK; INFN Sezione di Pavia, Pavia, Italy; Dipartimento di Fisica, Università di Pavia, Pavia, Italy; Department of Physics, University of Pennsylvania, Philadelphia, PA USA; Petersburg Nuclear Physics Institute, Gatchina, Russia; INFN Sezione di Pisa, Pisa, Italy; Dipartimento di Fisica E. Fermi, Università di Pisa, Pisa, Italy; Department of Physics and Astronomy, University of Pittsburgh, Pittsburgh, PA USA; Laboratorio de Instrumentacao e Fisica Experimental de Particulas-LIP, Lisbon, Portugal; Faculdade de Ciências, Universidade de Lisboa, Lisbon, Portugal; Department of Physics, University of Coimbra, Coimbra, Portugal; Centro de Física Nuclear da Universidade de Lisboa, Lisbon, Portugal; Departamento de Fisica, Universidade do Minho, Braga, Portugal; Departamento de Fisica Teorica y del Cosmos and CAFPE, Universidad de Granada, Granada, Spain; Dep Fisica and CEFITEC of Faculdade de Ciencias e Tecnologia, Universidade Nova de Lisboa, Caparica, Portugal; Institute of Physics, Academy of Sciences of the Czech Republic, Prague, Czech Republic; Czech Technical University in Prague, Prague, Czech Republic; Faculty of Mathematics and Physics, Charles University in Prague, Prague, Czech Republic; State Research Center Institute for High Energy Physics, Protvino, Russia; Particle Physics Department, Rutherford Appleton Laboratory, Didcot, UK; Ritsumeikan University, Kusatsu, Shiga Japan; INFN Sezione di Roma, Rome, Italy; Dipartimento di Fisica, Sapienza Università di Roma, Rome, Italy; INFN Sezione di Roma Tor Vergata, Rome, Italy; Dipartimento di Fisica, Università di Roma Tor Vergata, Rome, Italy; INFN Sezione di Roma Tre, Rome, Italy; Dipartimento di Matematica e Fisica, Università Roma Tre, Rome, Italy; Faculté des Sciences Ain Chock, Réseau Universitaire de Physique des Hautes Energies-Université Hassan II, Casablanca, Morocco; Centre National de l’Energie des Sciences Techniques Nucleaires, Rabat, Morocco; Faculté des Sciences Semlalia, Université Cadi Ayyad, LPHEA-Marrakech, Marrakech, Morocco; Faculté des Sciences, Université Mohamed Premier and LPTPM, Oujda, Morocco; Faculté des Sciences, Université Mohammed V-Agdal, Rabat, Morocco; DSM/IRFU (Institut de Recherches sur les Lois Fondamentales de l’Univers), CEA Saclay (Commissariat à l’Energie Atomique et aux Energies Alternatives), Gif-sur-Yvette, France; Santa Cruz Institute for Particle Physics, University of California Santa Cruz, Santa Cruz, CA USA; Department of Physics, University of Washington, Seattle, WA USA; Department of Physics and Astronomy, University of Sheffield, Sheffield, UK; Department of Physics, Shinshu University, Nagano, Japan; Fachbereich Physik, Universität Siegen, Siegen, Germany; Department of Physics, Simon Fraser University, Burnaby, BC Canada; SLAC National Accelerator Laboratory, Stanford, CA USA; Faculty of Mathematics, Physics and Informatics, Comenius University, Bratislava, Slovak Republic; Department of Subnuclear Physics, Institute of Experimental Physics of the Slovak Academy of Sciences, Kosice, Slovak Republic; Department of Physics, University of Cape Town, Cape Town, South Africa; Department of Physics, University of Johannesburg, Johannesburg, South Africa; School of Physics, University of the Witwatersrand, Johannesburg, South Africa; Department of Physics, Stockholm University, Stockholm, Sweden; The Oskar Klein Centre, Stockholm, Sweden; Physics Department, Royal Institute of Technology, Stockholm, Sweden; Departments of Physics and Astronomy and Chemistry, Stony Brook University, Stony Brook, NY USA; Department of Physics and Astronomy, University of Sussex, Brighton, UK; School of Physics, University of Sydney, Sydney, Australia; Institute of Physics, Academia Sinica, Taipei, Taiwan; Department of Physics, Technion: Israel Institute of Technology, Haifa, Israel; Raymond and Beverly Sackler School of Physics and Astronomy, Tel Aviv University, Tel Aviv, Israel; Department of Physics, Aristotle University of Thessaloniki, Thessaloniki, Greece; International Center for Elementary Particle Physics and Department of Physics, The University of Tokyo, Tokyo, Japan; Graduate School of Science and Technology, Tokyo Metropolitan University, Tokyo, Japan; Department of Physics, Tokyo Institute of Technology, Tokyo, Japan; Department of Physics, University of Toronto, Toronto, ON Canada; TRIUMF, Vancouver, BC, Canada; Department of Physics and Astronomy, York University, Toronto, ON Canada; Faculty of Pure and Applied Sciences, University of Tsukuba, Tsukuba, Japan; Department of Physics and Astronomy, Tufts University, Medford, MA USA; Centro de Investigaciones, Universidad Antonio Narino, Bogota, Colombia; Department of Physics and Astronomy, University of California Irvine, Irvine, CA USA; INFN Gruppo Collegato di Udine, Sezione di Trieste, Udine, Italy; ICTP, Trieste, Italy; Dipartimento di Chimica, Fisica e Ambiente, Università di Udine, Udine, Italy; Department of Physics, University of Illinois, Urbana, IL USA; Department of Physics and Astronomy, University of Uppsala, Uppsala, Sweden; Instituto de Física Corpuscular (IFIC) and Departamento de Física Atómica, Molecular y Nuclear and Departamento de Ingeniería Electrónica and Instituto de Microelectrónica de Barcelona (IMB-CNM), University of Valencia and CSIC, Valencia, Spain; Department of Physics, University of British Columbia, Vancouver, BC Canada; Department of Physics and Astronomy, University of Victoria, Victoria, BC Canada; Department of Physics, University of Warwick, Coventry, UK; Waseda University, Tokyo, Japan; Department of Particle Physics, The Weizmann Institute of Science, Rehovot, Israel; Department of Physics, University of Wisconsin, Madison, WI USA; Fakultät für Physik und Astronomie, Julius-Maximilians-Universität, Würzburg, Germany; Fachbereich C Physik, Bergische Universität Wuppertal, Wuppertal, Germany; Department of Physics, Yale University, New Haven, CT USA; Yerevan Physics Institute, Yerevan, Armenia; Centre de Calcul de l’Institut National de Physique Nucléaire et de Physique des Particules (IN2P3), Villeurbanne, France; CERN, 1211 Geneva 23, Switzerland

## Introduction

The mass of the top quark ($m_{\mathrm {top}}$) is an important parameter of the Standard Model (SM) of particle physics. Precise measurements of $m_{\mathrm {top}}$ provide critical inputs to fits of global electroweak parameters [1–3] that help assess the internal consistency of the SM. In addition, the value of $m_{\mathrm {top}}$ affects the stability of the SM Higgs potential, which has cosmological implications [4–6].

Many measurements of $m_{\mathrm {top}}$ were performed by the CDF and D0 collaborations based on Tevatron proton–antiproton collision data corresponding to integrated luminosities of up to 9.7  fb$^{-1}$. A selection of these measurements was used in the recent Tevatron $m_{\mathrm {top}}$ combination resulting in $m_{\mathrm {top}} = 174.34 \pm 0.37 \text{(stat) } \pm 0.52 \text{(syst) } {\mathrm { GeV}}= 174.34 \pm 0.64$ ${\mathrm { GeV}}$ [7]. Since 2010, measurements of $m_{\mathrm {top}}$ from the LHC by the ATLAS and CMS collaborations have become available. They are based on proton–proton (*pp*) collisions at a centre-of-mass energy of $\sqrt{s} = 7~{\mathrm { TeV}}$, recorded during 2010 and 2011 for integrated luminosities of up to 4.9 fb$^{-1}$ [8–13]. The corresponding LHC combination, based on $\sqrt{s} = 7~{\mathrm { TeV}}$ data and including preliminary results, yields $m_{\mathrm {top}} = 173.29 \pm 0.23 \text{(stat) } \pm 0.92 \text{(syst) } {\mathrm { GeV}}= 173.29 \pm 0.95$ ${\mathrm { GeV}}$ [14]. Using the same LHC input measurements and a selection of the $m_{\mathrm {top}}$ results from the Tevatron experiments, the first Tevatron$+$LHC $m_{\mathrm {top}}$ combination results in $m_{\mathrm {top}} = 173.34 \pm 0.27 \text{(stat) } \pm 0.71 \text{(syst) }$ GeV, with a total uncertainty of 0.76 ${\mathrm { GeV}}$ [15]. Recently, improved individual measurements with a total uncertainty compatible with that achieved in the Tevatron$+$LHC $m_{\mathrm {top}}$ combination have become available; the most precise single measurement is obtained by the D0 Collaboration using $t\bar{t}\rightarrow \text{ lepton+jets }$ events and yields $m_{\mathrm {top}} = 174.98\pm 0.76~{\mathrm { GeV}}$ [16].

This article presents a measurement of $$m_{\mathrm {top}} $$ using events with one or two isolated charged leptons (electrons or muons) in the final state (the $$t\bar{t}\rightarrow \text{ lepton+jets } $$ and $$t\bar{t}\rightarrow \text{ dilepton } $$ decay channels), in $$4.6 $$ $$\text{ fb }^{-1}$$ of *pp* collision data collected by the ATLAS detector at a centre-of-mass energy of $$\sqrt{s} =7$$ $${\mathrm { TeV}}$$ during 2011. It supersedes Ref. [8], where, using a two-dimensional fit to reconstructed observables in the $$t\bar{t}\rightarrow \text{ lepton+jets } $$ channel, $$m_{\mathrm {top}} $$ was determined together with a global jet energy scale factor. The use of this scale factor allows the uncertainty on $$m_{\mathrm {top}} $$ stemming from imperfect knowledge of the jet energy scale (JES) to be considerably reduced, albeit at the cost of an additional statistical uncertainty component. The single largest systematic uncertainty on $$m_{\mathrm {top}} $$ in Ref. [8] was due to the relative *b*-to-light-jet energy scale (bJES) uncertainty, where the terms $$b\text{-jets } $$ and light-jets refer to jets originating from $$b\text{-quarks } $$ and *u*, *d*, *c*, *s*-quarks or gluons, respectively. To reduce this uncertainty in the present analysis, a three-dimensional template fit is used for the first time in the $$t\bar{t}\rightarrow \text{ lepton+jets } $$ channel, again replacing the corresponding uncertainty by a statistical uncertainty and a reduced systematic uncertainty. This concept will be even more advantageous with increasing data luminosity. In addition, for the combination of the measurements of $$m_{\mathrm {top}} $$ in the two decay channels an in-depth investigation of the correlation of the two estimators for all components of the sources of systematic uncertainty is made. This leads to a much smaller total correlation of the two measurements than what is typically assigned, such that their combination yields a very significant improvement in the total uncertainty on $$m_{\mathrm {top}} $$. To retain this low correlation, the jet energy scale factors measured in the $$t\bar{t}\rightarrow \text{ lepton+jets } $$ channel have not been propagated to the $$t\bar{t}\rightarrow \text{ dilepton } $$ channel.

In the $t\bar{t}\rightarrow \text{ lepton+jets }$ channel, one *W* boson from the top or antitop quark decays directly or via an intermediate $\tau$ decay into an electron or muon and at least one neutrino, while the other *W* boson decays into a quark–antiquark pair. The $t\bar{t}$ decay channels with electrons and muons are combined and referred to as the lepton$+$jets (or as a shorthand $\ell \text{+jets }$) final state. The $t\bar{t}\rightarrow \text{ dilepton }$ channel corresponds to the case where both *W* bosons from the top and antitop quarks decay leptonically, directly or via an intermediate $\tau$ decay, into an electron or muon and at least one neutrino. The $t\bar{t}$ decay channels $ee, e\mu , \mu \mu$ are combined and referred to as the $\text{ dilepton }$ final state. For both the $\ell \text{+jets }$ and $\text{ dilepton }$ final states, the measurements are based on the template method [17]. In this technique, Monte Carlo (MC) simulated distributions are constructed for a chosen quantity sensitive to the physics parameter under study, using a number of discrete values of that parameter. These templates are fitted to analytical functions that interpolate between different input values of the physics parameter, fixing all other parameters of the functions. In the final step a likelihood fit to the observed distribution in data is used to obtain the value for the physics parameter that best describes the data. In this procedure the top quark mass determined from data corresponds to the mass definition used in the MC simulation. It is expected that the difference between this mass definition and the pole mass is of order 1 GeV [18–21].

In the $\ell \text{+jets }$ channel, events are reconstructed using a kinematic fit that assumes a $t\bar{t}$ topology. A three-dimensional template method is used, where $m_{\mathrm {top}}$ is determined simultaneously with a light-jet energy scale factor ($\text{ JSF }$), exploiting the information from the hadronic *W* decays, and a separate *b*-to-light-jet energy scale factor ($\text{ bJSF }$). The $\text{ JSF }$ and $\text{ bJSF }$ account for residual differences of data and simulation in the light-jet and in the relative *b*-to-light-jet energy scale, respectively, thereby mitigating the corresponding systematic uncertainties on $m_{\mathrm {top}}$. The analysis in the $\text{ dilepton }$ channel is based on a one-dimensional template method, where the templates are constructed for the $m_{\ell b}$ observable, defined as the per-event average invariant mass of the two lepton$-b$-jet systems from the decay of the top quarks. Due to the underconstrained kinematics associated with the $\text{ dilepton }$ final state, no in situ constraint of the jet energy scales is performed.

This article is organised as follows: after a short description of the ATLAS detector in Sect. 2, the data and MC simulation samples are discussed in Sect. 3. Details of the event selection and reconstruction are given in Sect. 4. The template fits are explained in Sect. 5. The measurement of $m_{\mathrm {top}}$ in the two final states is given in Sect. 6, and the evaluation of the associated systematic uncertainties are discussed in Sect. 7. The results of the combination of the $m_{\mathrm {top}}$ measurements from the individual analyses are reported in Sect. 8. Finally, the summary and conclusions are given in Sect. 9.

## The ATLAS detector

The ATLAS detector [22] covers nearly the entire solid angle around the collision point.^1^ It consists of an inner tracking detector surrounded by a thin superconducting solenoid, electromagnetic and hadronic calorimeters, and a muon spectrometer incorporating three large superconducting toroid magnets. The inner-detector system (ID) is immersed in a 2 T axial magnetic field and provides charged-particle tracking in the range $|\eta | < 2.5$. The high-granularity silicon pixel detector covers the interaction region and typically provides three measurements per track, the first energy deposit being normally in the innermost layer. It is followed by the silicon microstrip tracker designed to provide four two-dimensional measurement points per track. These silicon detectors are complemented by the transition radiation tracker, which enables radially extended track reconstruction up to $|\eta | = 2.0$. The transition radiation tracker also provides electron identification information based on the fraction of energy deposits (typically 30 hits in total) above an energy threshold corresponding to transition radiation. The calorimeter system covers the pseudorapidity range $|\eta | < 4.9$. Within the region $|\eta |< 3.2$, electromagnetic calorimetry is provided by barrel and endcap high-granularity lead/liquid-argon (LAr) electromagnetic calorimeters, with an additional thin LAr presampler covering $|\eta | < 1.8$, to correct for energy loss in material upstream of the calorimeters. Hadronic calorimetry is provided by the steel/scintillator-tile calorimeter, segmented into three barrel structures within $|\eta | < 1.7$, and two copper/LAr hadronic endcap calorimeters. The solid angle coverage is completed with forward copper/LAr and tungsten/LAr calorimeter modules optimised for electromagnetic and hadronic measurements respectively. The muon spectrometer (MS) comprises separate trigger and high-precision tracking chambers measuring the deflection of muons in the magnetic field generated by the toroids. The precision chamber system covers the region $|\eta | < 2.7$ with three layers of monitored drift tubes, complemented by cathode strip chambers in the forward region. The muon trigger system covers the range $|\eta | < 2.4$ with resistive plate chambers in the barrel, and thin gap chambers in the endcap regions. A three-level trigger system is used to select interesting events [23]. The Level-1 trigger is implemented in hardware and uses a subset of detector information to reduce the event rate to at most 75 kHz. This is followed by two software-based trigger levels which together reduce the event rate to about 300 Hz.

## Data and Monte Carlo samples

For the measurements described in this document, data from LHC *pp* collisions at $$\sqrt{s} =7$$ $${\mathrm { TeV}}$$ are used. They correspond to an integrated luminosity of $$4.6 $$ $$\text{ fb }^{-1}$$ with an uncertainty of $$1.8~\% $$ [24], and were recorded during 2011 during stable beam conditions and with all relevant ATLAS sub-detector systems operational.

MC simulations are used to model $t\bar{t}$ and single top quark processes as well as some of the background contributions. Top quark pair and single top quark production (in the *s*- and *Wt*-channels) are simulated using the next-to-leading-order (NLO) MC program Powheg-hvq (patch4) [25] with the NLO CT10 [26] parton distribution functions (PDFs). Parton showering, hadronisation and the underlying event are modelled using the Pythia (v6.425) [27] program with the Perugia 2011C (P2011C) MC parameter set (tune) [28] and the corresponding CTEQ6L1 PDFs [29]. The AcerMC (v3.8) generator [30] interfaced with Pythia (v6.425) is used for the simulation of the single top quark *t*-channel process. The AcerMC and Pythia programs are used with the CTEQ6L1 PDFs and the corresponding P2011C tune.

For the construction of signal templates, the $$t\bar{t}$$ and single top quark production samples are generated for different assumed values of $$m_{\mathrm {top}} $$, namely $$167.5, 170, 172.5, 175, 177.5~{\mathrm { GeV}}$$. The $$t\bar{t}$$ MC samples are normalised to the predicted $$t\bar{t}$$ cross section for each $$m_{\mathrm {top}} $$ value. The $$t\bar{t}$$ cross section for *pp* collisions at $$\sqrt{s} = 7 {\mathrm { TeV}}$$ is $$\sigma _{t\bar{t}}= 177^{+10}_{-11}$$ pb for $$m_{\mathrm {top}} =172.5$$ $${\mathrm { GeV}}$$. It was calculated at next-to-next-to-leading-order (NNLO) in QCD including resummation of next-to-next-to-leading-logarithmic (NNLL) soft gluon terms with Top$$++$$2.0 [31–36]. The PDF$$+$$$$\alpha _{s} $$ uncertainties on the cross section were calculated using the PDF4LHC prescription [37] with the MSTW2008 $$68\,\%$$ CL NNLO [38, 39], CT10 NNLO [26, 40] and NNPDF2.3 5f FFN [41] PDFs, and added in quadrature to the factorisation and renormalisation scale uncertainty. The NNLO$$+$$NNLL value, as implemented in Hathor 1.5 [42], is about $$3\,\%$$ larger than the plain NNLO prediction. The single top quark production cross sections are normalised to the approximate NNLO prediction values. For example, for $$m_{\mathrm {top}} =172.5$$ $${\mathrm { GeV}}$$, these are $$64.6^{+2.7}_{-2.0}$$ pb [43], $$4.6\pm 0.2$$ pb [44] and $$15.7\pm 1.1$$ pb [45] for the *t*-, *s*- and *Wt*-production channels respectively.

The production of $$W $$ or $$Z $$ bosons in association with jets is simulated using the Alpgen (v2.13) generator [46] interfaced to the Herwig (v6.520) [47, 48] and Jimmy (v4.31) [49] packages. The CTEQ6L1 PDFs and the corresponding AUET2 tune [50] are used for the matrix element and parton shower settings. The $$W+$$jets events containing heavy-flavour quarks ($$Wbb+$$jets, $$Wcc+$$jets, and $$Wc+$$jets) are generated separately using leading-order matrix elements with massive *b*- and $$c\text{-quarks } $$. An overlap-removal procedure is used to avoid double counting of heavy-flavour quarks between the matrix element and the parton shower evolution. Diboson production processes (*WW*, *WZ* and *ZZ*) are produced using the Herwig generator with the AUET2 tune.

Multiple *pp* interactions generated with Pythia (v6.425) using the AMBT2B tune [51] are added to all MC samples. These simulated events are re-weighted such that the distribution of the number of interactions per bunch crossing (pile-up) in the simulated samples matches that in the data. The average number of interactions per bunch crossing for the data set considered is 8.7. The samples are processed through a simulation of the ATLAS detector [52] based on GEANT4 [53] and through the same reconstruction software as the data.

## Event selection and reconstruction

### Object selection

In this analysis $t\bar{t}$ events with one or two isolated charged leptons in the final states are selected. The event selection for both final states is based on the following reconstructed objects in the detector: electron and muon candidates, jets and missing transverse momentum ($E_{\text {T}}^{\text {miss}}$).

An electron candidate is defined as an energy deposit in the electromagnetic calorimeter with an associated well-reconstructed track [54]. Electron candidates are required to have transverse energy $$E_{\text {T}} >25$$ $${\mathrm { GeV}}$$ and $$\vert \eta _\mathrm {cluster} \vert < 2.47$$, where $$\eta _\mathrm {cluster} $$ is the pseudorapidity of the electromagnetic cluster associated with the electron. Candidates in the transition region between the barrel and endcap calorimeter ($$1.37<\vert \eta _\mathrm {cluster} \vert <1.52$$) are excluded. Muon candidates are reconstructed from track segments in different layers of the MS [55]. These segments are combined starting from the outermost layer, with a procedure that takes effects of detector material into account, and matched with tracks found in the ID. The final candidates are refitted using the complete track information, and are required to satisfy $$p_{\text {T}} >20$$ $${\mathrm { GeV}}$$ and $$\vert \eta \vert <2.5$$. Isolation criteria, which restrict the amount of energy deposited near the lepton candidates, are applied to both the electrons and muons to reduce the backgrounds from heavy-flavour decays inside jets or photon conversions, and the background from hadrons mimicking lepton signatures, in the following referred to as non-prompt and fake-lepton background (NP/fake-lepton background). For electrons, the energy not associated with the electron cluster and contained in a cone of $$\Delta R = 0.2$$ around the electron must not exceed an $$\eta $$-dependent threshold ranging from 1.25 to 3.7 $${\mathrm { GeV}}$$. Similarly, the total transverse momentum of the tracks contained in a cone of $$\Delta R=0.3$$ must not exceed a threshold ranging from 1.00 to 1.35 $${\mathrm { GeV}}$$, depending on the electron candidate $$p_{\text {T}} $$ and $$\eta $$. For muons, the sum of track transverse momenta in a cone of $$\Delta R=0.3$$ around the muon is required to be less than 2.5 $${\mathrm { GeV}}$$, and the total energy deposited in a cone of $$\Delta R=0.2$$ around the muon is required to be less than $$4 $$ $${\mathrm { GeV}}$$. The longitudinal impact parameter of each charged lepton along the beam axis is required to be within 2 mm of the reconstructed primary vertex, defined as the vertex with the highest $$\sum _\mathrm{trk} p_\mathrm{T,trk}^2$$, among all candidates with at least five associated tracks with $$p_\mathrm{T,trk} > 0.4~{\mathrm { GeV}}$$.

Jets are reconstructed with the anti-$k_\mathrm {t}$ algorithm [56] using a radius parameter of $R=0.4$, starting from energy clusters of adjacent calorimeter cells called topological clusters [57]. These jets are calibrated first by correcting the jet energy using the scale established for electromagnetic objects (EM scale). They are further corrected to the hadronic energy scale using calibration factors that depend on the jet energy and $\eta$, obtained from simulation. Finally, a residual in situ calibration derived from both data and MC simulation is applied [58]. Jet quality criteria are applied to identify and reject jets reconstructed from energy deposits in the calorimeters originating from particles not emerging from the bunch crossing under study [59]. To suppress the contribution from low-$p_{\text {T}}$ jets originating from pile-up interactions, tracks associated with the jet and emerging from the primary vertex are required to account for at least 75 % of the scalar sum of the $p_{\text {T}}$ of all tracks associated with the jet. Jets with no associated tracks are also accepted.

Muons reconstructed within a $\Delta R=0.4$ cone around a jet satisfying $p_{\text {T}} >25$ ${\mathrm { GeV}}$ are removed to reduce the contamination caused by muons from hadron decays within jets. Subsequently, jets within a $\Delta R=0.2$ cone around an electron candidate are removed to avoid double counting, which can occur because electron clusters are usually also reconstructed as jets. After this jet overlap removal, electrons are rejected if their distance to the closest jet is smaller than $\Delta R=0.4$.

The reconstruction of $E_{\text {T}}^{\text {miss}}$ is based on the vector sum of calorimeter energy deposits projected onto the transverse plane. The $E_{\text {T}}^{\text {miss}}$ is reconstructed from topological clusters, calibrated at the EM scale and corrected according to the energy scale of the corresponding identified physics objects. Contributions from muons are included by using their momentum as measured by the inner detector and muon spectrometer [60].

The reconstruction of top quark pair events is facilitated by the ability to tag jets originating from $b\text{-quarks }$. For this purpose the neural-network-based MV1 algorithm is applied [61, 62]. In the following, irrespective of their origin, jets tagged by this algorithm are called $b\text{-tagged }$ jets, whereas those not tagged are called untagged jets. Similarly, whether they are tagged or not, jets originating from $b\text{-quarks }$ and from *u*, *d*, *c*, *s*-quarks or gluons are called $b\text{-jets }$ and light-jets, respectively. The MV1 algorithm relies on track impact parameters and the properties of reconstructed secondary vertices such as the decay length significance. The chosen working point corresponds to a $b\text{-tagging }$ efficiency of 75 % for $b\text{-jets }$ in simulated $t\bar{t}$ events and a light-jet (*c*-quark jet) rejection factor of about 60 (4). To match the $b\text{-tagging }$ performance in the data, $p_{\text {T}}$- and $\eta$-dependent scale factors are applied to MC jets depending on their original flavour. The scale factors are obtained from dijet [62] and $t\bar{t}\rightarrow \text{ dilepton }$ events. The $t\bar{t}$-based calibration is obtained using the methodology described in Ref. [63], applied to the 7 ${\mathrm { TeV}}$ data. The scale factors are calculated per jet and finally multiplied to obtain an event weight for any reconstructed distribution.

### Event selection

The $$t\bar{t}\rightarrow \text{ lepton+jets } $$ signal is characterised by an isolated charged lepton with relatively high $$p_{\text {T}} $$, $$E_{\text {T}}^{\text {miss}} $$ arising from the neutrino from the leptonic $$W $$ boson decay, two $$b\text{-jets } $$ and two light-jets from the hadronic $$W $$ boson decay. The main contributions to the background stem from $$W \text{+jets } $$ production and from the NP/fake-lepton background. The normalisation of the $$W \text{+jets } $$ background is estimated from data, based on the charge-asymmetry method [64], and the shape is obtained from simulation. For the NP/fake-lepton background, both the shape of the distributions and the normalisation are estimated from data by weighting each selected event by the probability of containing a NP/fake lepton. This contribution in both the electron and the muon channel is estimated using a data-driven matrix method based on selecting two categories of events, using loose and tight lepton selection requirements [65]. The contributions from single top quark, $$Z+$$jets, and diboson production are taken from simulation, normalised to the best available theoretical cross sections.

The $$t\bar{t}\rightarrow \text{ dilepton } $$ events are characterised by the presence of two isolated and oppositely charged leptons with relatively high $$p_{\text {T}} $$, $$E_{\text {T}}^{\text {miss}} $$ arising from the neutrinos from the leptonic $$W $$ boson decays, and two $$b\text{-jets } $$. Background processes with two charged leptons from $$W $$- or $$Z $$ decays in the final state, which are similar to the $$t\bar{t}\rightarrow \text{ dilepton } $$ events, are dominated by single top quark production in the *Wt*-channel. Additional contributions come from $$Z+$$jets processes and diboson production with additional jets. In the analysis, these contributions are estimated directly from the MC simulation normalised to the relevant cross sections. Events may also be wrongly reconstructed as $$t\bar{t}\rightarrow \text{ dilepton } $$ events due to the presence of NP/fake leptons together with $$b\text{-tagged } $$ jets and $$E_{\text {T}}^{\text {miss}} $$. As for the $$t\bar{t}\rightarrow \text{ lepton+jets } $$ channel, the NP/fake-lepton background is estimated using a data-driven matrix method [65].

The selection of $$t\bar{t}$$ event candidates consists of a series of requirements on the general event quality and the reconstructed objects designed to select events consistent with the above signal topologies. To suppress non-collision background, events are required to have at least one good primary vertex. It is required that the appropriate single-electron or single-muon trigger has fired; the trigger thresholds are 20 or 22 $${\mathrm { GeV}}$$ (depending on the data-taking period) for the electrons and 18 $${\mathrm { GeV}}$$ for muons. Candidate events in the $$\ell \text{+jets } $$ final state are required to have exactly one reconstructed charged lepton with $$E_{\text {T}} > 25$$ $${\mathrm { GeV}}$$ for electrons, and $$p_{\text {T}} > 20$$ $${\mathrm { GeV}}$$ for muons, matching the corresponding trigger object. Exactly two oppositely charged leptons, with at least one matching a trigger object, are required in the $$\text{ dilepton } $$ final state. In the $$\mu \text{+jets } $$ channel, $$E_{\text {T}}^{\text {miss}} >20$$ $${\mathrm { GeV}}$$ and $$E_{\text {T}}^{\text {miss}} +m_{\mathrm {T}}^{W} >60$$ $${\mathrm { GeV}}$$ are required.^2^ In the $$e\text{+jets } $$ channel more stringent selections on $$E_{\text {T}}^{\text {miss}} $$ and $$m_{\mathrm {T}}^{W} $$ ($$E_{\text {T}}^{\text {miss}} > 30$$ $${\mathrm { GeV}}$$ and $$m_{\mathrm {T}}^{W} >30$$ $${\mathrm { GeV}}$$) are imposed due to the higher level of NP/fake-lepton background. For the *ee* and $$\mu \mu $$ channels, in the $$\text{ dilepton } $$ final state, $$E_{\text {T}}^{\text {miss}} >60~{\mathrm { GeV}}$$ is required. In addition, the invariant mass of the same-flavour charged-lepton pair, $$m_{\ell \ell }$$ $$(\ell \ell = ee, \mu \mu )$$, is required to exceed 15 $${\mathrm { GeV}}$$, to reduce background from low-mass resonances decaying into charged lepton–antilepton pairs and Drell–Yan production. Similarly, to reduce the $$Z+$$jets background, values of $$m_{\ell \ell }$$ compatible with the $$Z $$ boson mass are vetoed by requiring $$|m_{\ell \ell } - 91~{\mathrm { GeV}}| > 10~{\mathrm { GeV}}$$. In the $$e\mu $$ channel $$H_{\mathrm {T}} >130~{\mathrm { GeV}}$$ is required, where $$H_{\mathrm {T}} $$ is the scalar sum of the $$p_{\text {T}} $$ of the two selected charged leptons and the jets. Finally, the event is required to have at least four jets (or at least two jets for the $$t\bar{t}\rightarrow \text{ dilepton } $$ channel) with $$p_{\text {T}} >25$$ $${\mathrm { GeV}}$$ and $$\vert \eta \vert <2.5$$. At least one of these jets must be $$b\text{-tagged } $$ for the $$t\bar{t}\rightarrow \text{ lepton+jets } $$ analysis. In the $$\text{ dilepton } $$ final state, events are accepted if they contain exactly one or two $$b\text{-tagged } $$ jets.

These requirements select 61786 and 6661 data events in the $t\bar{t}\rightarrow \text{ lepton+jets }$ and $t\bar{t}\rightarrow \text{ dilepton }$ channels, with expected background fractions of 22 % and 2 %, respectively. Due to their inherent $m_{\mathrm {top}}$ sensitivity, here and in the following, the single top quark processes are accounted for as signal in both analyses, and not included in the quoted background fractions.

### Event reconstruction

After the event selection described in the previous section, the events are further reconstructed according to the decay topology of interest, and are subject to additional requirements.

#### Kinematic reconstruction of the lepton$+$jets final state

A kinematic likelihood fit [8, 66] is used to fully reconstruct the $t\bar{t}\rightarrow \text{ lepton+jets }$ kinematics. The algorithm relates the measured kinematics of the reconstructed objects to the leading-order representation of the $t\bar{t}$ system decay. The event likelihood is constructed as the product of Breit–Wigner (BW) distributions and transfer functions (TF). The *W* boson BW line-shape functions use the world combined values of the *W* boson mass and decay width from Ref. [3]. A common mass parameter, $m_{\mathrm {top}} ^{\mathrm {reco}}$, is used for the BW distributions describing the leptonically and hadronically decaying top quarks, and this is fitted event-by-event. The top quark width varies with $m_{\mathrm {top}} ^{\mathrm {reco}}$ and it is calculated according to the SM prediction [3]. The TF are derived from the Powheg$+$Pythia$t\bar{t}$ signal MC simulation sample at an input mass of $m_{\mathrm {top}} =172.5$ ${\mathrm { GeV}}$. They represent the experimental resolutions in terms of the probability that the observed energy at reconstruction level is produced by a given parton-level object for the leading-order decay topology.

The input objects to the likelihood are: the reconstructed charged lepton, the missing transverse momentum and four jets. For the sample with one $$b\text{-tagged } $$ jet these are the $$b\text{-tagged } $$ jet and the three untagged jets with the highest $$p_{\text {T}} $$. For the sample with at least two $$b\text{-tagged } $$ jets these are the two highest-$$p_{\text {T}} $$$$b\text{-tagged } $$ jets, and the two highest-$$p_{\text {T}} $$ remaining jets. The *x*- and *y*-components of the missing transverse momentum are used as starting values for the neutrino transverse momentum components, with its longitudinal component ($$p_{\nu ,z}$$) as a free parameter in the kinematic likelihood fit. Its starting value is computed from the $$W\rightarrow \ell \nu $$ mass constraint. If there are no real solutions for $$p_{\nu ,z}$$ a starting value of zero is used. If there are two real solutions, the one giving the largest likelihood value is taken.

Maximising the event-by-event likelihood as a function of $m_{\mathrm {top}} ^{\mathrm {reco}}$ establishes the best assignment of reconstructed jets to partons from the $t\bar{t}\rightarrow \text{ lepton+jets }$ decay. The maximisation is performed by testing all possible permutations, assigning jets to partons. The likelihood is extended by including the probability for a jet to be $b\text{-tagged }$, given the parton from the top quark decay it is associated with, to construct an event probability. The *b*-tagging efficiencies and rejection factors are used to favour permutations for which a $b\text{-tagged }$ jet is assigned to a *b*-quark and penalise those where a $b\text{-tagged }$ jet is assigned to a light quark. The permutation of jets with the highest likelihood value is retained.

The value of $$m_{\mathrm {top}} ^{\mathrm {reco}} $$ obtained from the kinematic likelihood fit is used as the observable primarily sensitive to the underlying $$m_{\mathrm {top}} $$. The invariant mass of the hadronically decaying $$W $$ boson ($$m_{W}^{\mathrm {reco}} $$) is calculated from the assigned jets of the chosen permutation. Finally, an observable called $$R_{b q} ^{\mathrm {reco}} $$, designed to be sensitive to the relative *b*-to-light-jet energy scale, is computed in the following way. For events with only one $$b\text{-tagged } $$ jet, $$R_{b q} ^{\mathrm {reco}} $$ is defined as the ratio of the transverse momentum of the $$b\text{-tagged } $$ jet to the average transverse momentum of the two jets of the hadronic $$W $$ boson decay. For events with two or more $$b\text{-tagged } $$ jets, $$R_{b q} ^{\mathrm {reco}} $$ is defined as the scalar sum of the transverse momenta of the $$b\text{-tagged } $$ jets assigned to the leptonically and hadronically decaying top quarks divided by the scalar sum of the transverse momenta of the two jets associated with the hadronic $$W $$ boson decay. The values of $$m_{W}^{\mathrm {reco}} $$ and $$R_{b q} ^{\mathrm {reco}} $$ are computed from the jet four-vectors as given by the jet reconstruction to keep the maximum sensitivity to changes of the jet energy scale for light-jets and $$b\text{-jets } $$.

In view of the template parameterisation described in Sect. 5 additional selection criteria are applied. Events in which a $$b\text{-tagged } $$ jet is assigned to the $$W $$ decay by the likelihood fit are discarded. This is needed to prevent mixing effects between the information provided by the $$m_{W}^{\mathrm {reco}} $$ and $$R_{b q} ^{\mathrm {reco}} $$ distributions. The measured $$m_{\mathrm {top}} ^{\mathrm {reco}} $$ is required to be in the range 125–225 $${\mathrm { GeV}}$$ for events with one $$b\text{-tagged } $$ jet, and in the range 130–220 $${\mathrm { GeV}}$$ for events with at least two $$b\text{-tagged } $$ jets. In addition, $$m_{W}^{\mathrm {reco}} $$ is required to be in the range 55–110 $${\mathrm { GeV}}$$ and finally, $$R_{b q} ^{\mathrm {reco}} $$ is required to be in the range 0.3–3.0. The fraction of data events which pass these requirements is 35 %. Although removing a large fraction of data, these requirements remove events in the tails of the three distributions, which are typically poorly reconstructed with small likelihood values and do not contain significant information on $$m_{\mathrm {top}} $$. In addition, the templates then have simpler shapes which are easier to model analytically with fewer parameters.

#### Reconstruction of the $\text{ dilepton }$ final state

In the $$t\bar{t}\rightarrow \text{ dilepton } $$ channel the kinematics are under-constrained due to the presence of at least two undetected neutrinos. Consequently, instead of attempting a full reconstruction, the $$m_{\mathrm {top}} $$-sensitive observable $$m_{\ell b} $$ is defined based on the invariant mass of the two charged-lepton$$+$$$$b\text{-jet } $$ pairs.

The preselected events contain two charged leptons, at least two jets, of which either exactly one or exactly two are $$b\text{-tagged } $$. For events with exactly two $$b\text{-tagged } $$ jets the charged-lepton$$+$$$$b\text{-tagged } $$ jet pairs can be built directly. In the case of events with only one $$b\text{-tagged } $$ jet the missing second $$b\text{-jet } $$ is identified with the untagged jet carrying the highest MV1 weight. For both classes of events, when using the two selected jets and the two charged leptons, there are two possible assignments for the jet-lepton pairs, each leading to two values for the corresponding pair invariant masses. The assignment resulting in the lowest average mass is retained, and this mass is taken as the $$m_{\ell b}^{\mathrm {reco}} $$ estimator of the event. The measured $$m_{\ell b}^{\mathrm {reco}} $$ is required to be in the range 30–170 $${\mathrm { GeV}}$$. This extra selection retains 97 % of the data candidate events.

#### Event yields

**Fig. 1:**
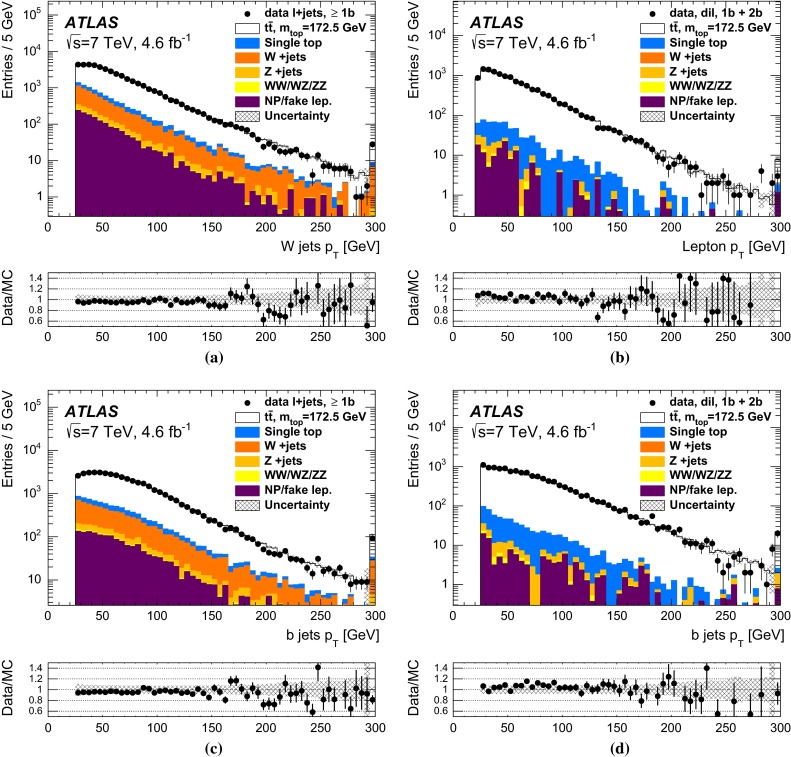
Distributions of the transverse momentum of the untagged and $b\text{-tagged }$ jets in the $t\bar{t}\rightarrow \text{ lepton+jets }$ analysis (**a**, **c**) and of the charged lepton and $b\text{-tagged }$ jets $p_{\text {T}}$ in the $t\bar{t}\rightarrow \text{ dilepton }$ analysis (**b**, **d**). The data are shown by the*points* and the signal-plus-background prediction by the *solid histogram*. The *hatched area* is the combined uncertainty on the prediction described in Sect. 4.3.3, and the *rightmost bin* contains the overflow if present. For each figure, the ratio of the data to the MC prediction is also presented

The numbers of events observed and expected after the above selections are reported in Table 1 for the $\ell \text{+jets }$ and $\text{ dilepton }$ final states. The observed numbers of events are well described by the sum of the signal and background estimates within uncertainties. The latter are estimated as the sum in quadrature of the statistical uncertainty, the uncertainty on the $b\text{-tagging }$ efficiencies, a $1.8~\%$ uncertainty on the integrated luminosity [24], the uncertainties on the $t\bar{t}$ and single top quark theoretical cross sections, a $30~\%$ uncertainty on the $W \text{+jets }$ and $Z \text{+jets }$ normalisation, and finally a $50~\%$ uncertainty on the NP/fake-lepton background normalisation. The distribution of several kinematic variables in the data were inspected and found to be well described by the signal-plus-background prediction, within uncertainties. As examples, Fig. 1 (left) shows the distribution of the untagged and $b\text{-tagged }$ jets $p_{\text {T}}$ observed in the $\ell \text{+jets }$ final state. Similarly, the $p_{\text {T}}$ distributions for the charged leptons and $b\text{-tagged }$ jets in the $\text{ dilepton }$ final state are shown on the right of Fig. 1. In all cases the data are compared with the MC predictions, assuming an input top quark mass of 172.5 ${\mathrm { GeV}}$.

**Table 1:** The observed numbers of events, according to the $$b\text{-tagged } $$ jet multiplicity, in the $$\ell \text{+jets } $$ and $$\text{ dilepton } $$ final states in $$4.6 $$ $$\text{ fb }^{-1}$$ of $$\sqrt{s} = 7$$ $${\mathrm { TeV}}$$ data. In addition, the expected numbers of signal and background events corresponding to the integrated luminosity of the data are given. The predictions are quoted using two significant digits for their uncertainty. The MC estimates assume SM cross sections. The $$W \text{+jets } $$ and NP/fake-lepton background contributions are estimated from data. The uncertainties for the estimates include the components detailed in Sect. 4.3.3. Values smaller than 0.005 are listed as 0.00

$\ell \text{+jets }$ final state			
Process	One $b\text{-tagged }$ jet	At least two $b\text{-tagged }$ jets	Sum
$t\bar{t}$ signal	9890 $\pm$ 630	8210 $\pm$ 560	18100 $\pm$ 1100
Single top quark (signal)	756 $\pm$ 41	296 $\pm$ 19	1052 $\pm$ 57
$W+$jets (data)	2250 $\pm$ 680	153 $\pm$ 49	2400 $\pm$ 730
$Z+$jets	284 $\pm$ 87	18.5 $\pm$ 6.1	303 $\pm$ 93
*WW* / *WZ* / *ZZ*	43.5 $\pm$ 2.3	4.65 $\pm$ 0.48	48.2 $\pm$ 2.6
NP/fake leptons (data)	700 $\pm$ 350	80 $\pm$ 41	780 $\pm$ 390
Signal$+$background	13920 $\pm$ 1000	8760 $\pm$ 560	22700 $\pm$ 1400
Data	12979	8784	21763
Exp. Bkg. frac.	0.25 $\pm$ 0.02	0.03 $\pm$ 0.00	0.16 $\pm$ 0.01
Data/MC	0.93 $\pm$ 0.07	1.00 $\pm$ 0.07	0.96 $\pm$ 0.06

Dilepton final state			
Process	One $b\text{-tagged }$ jet	Two $b\text{-tagged }$ jets	Sum
$t\bar{t}$ signal	2840 $\pm$ 180	2950 $\pm$ 210	5790 $\pm$ 360
Single top quark (signal)	181 $\pm$ 10	82.5 $\pm$ 5.7	264 $\pm$ 15
$Z+$jets	34 $\pm$ 11	4.1 $\pm$ 1.5	38 $\pm$ 12
*WW* / *WZ* / *ZZ*	7.01 $\pm$ 0.63	0.61 $\pm$ 0.15	7.62 $\pm$ 0.67
NP/fake leptons (data)	52 $\pm$ 28	2.6 $\pm$ 8.4	55 $\pm$ 30
Signal$+$background	3110 $\pm$ 180	3040 $\pm$ 210	6150 $\pm$ 360
Data	3227	3249	6476
Exp. Bkg. frac.	0.03 $\pm$ 0.00	0.00 $\pm$ 0.00	0.02 $\pm$ 0.00
Data/MC	1.04 $\pm$ 0.06	1.07 $\pm$ 0.07	1.05 $\pm$ 0.06

## Analysis method

The observables exploited in the $m_{\mathrm {top}}$ analyses are: $m_{\mathrm {top}} ^{\mathrm {reco}}$, $m_{W}^{\mathrm {reco}}$, $R_{b q} ^{\mathrm {reco}}$ in the $t\bar{t}\rightarrow \text{ lepton+jets }$ channel and $m_{\ell b}^{\mathrm {reco}}$ in the $t\bar{t}\rightarrow \text{ dilepton }$ channel.

In the $t\bar{t}\rightarrow \text{ lepton+jets }$ channel, templates of $m_{\mathrm {top}} ^{\mathrm {reco}}$ are constructed as a function of the top quark mass used in the MC generation in the range 167.5–177.5 ${\mathrm { GeV}}$, in steps of 2.5 ${\mathrm { GeV}}$. In addition, for the central mass point, templates of $m_{\mathrm {top}} ^{\mathrm {reco}}$ are constructed for an input value of the light-jet energy scale factor ($\text{ JSF }$) in the range 0.95–1.05 in steps of 2.5 % and for an input value for the relative *b*-to-light-jet energy scale factor ($\text{ bJSF }$) in the same range. Independent MC samples are used for the different $m_{\mathrm {top}}$ mass points, and from those samples templates with different values of $\text{ JSF }$ and $\text{ bJSF }$ are extracted by appropriately scaling the four-momentum of the jets in each sample. The input value for the $\text{ JSF }$ is applied to all jets, whilst the input value for the $\text{ bJSF }$ is applied to all $b\text{-jets }$ according to the information about the generated quark flavour. This scaling is performed after the various correction steps of the jet calibration and before any event selection. This results in different events entering the final selection from one energy scale variation to another. Similarly, templates of $m_{W}^{\mathrm {reco}}$ are constructed as a function of an input $\text{ JSF }$ combining the samples from all $m_{\mathrm {top}}$ mass points. Finally, templates of $R_{b q} ^{\mathrm {reco}}$ are constructed as a function of $m_{\mathrm {top}}$, and as a function of an input $\text{ bJSF }$ at the central mass point.

In the $t\bar{t}\rightarrow \text{ dilepton }$ channel, signal templates for $m_{\ell b}^{\mathrm {reco}}$ are constructed as a function of the top quark mass used in the MC generation in the range 167.5–177.5 ${\mathrm { GeV}}$, using separate samples for each of the five mass points.

**Fig. 2:**
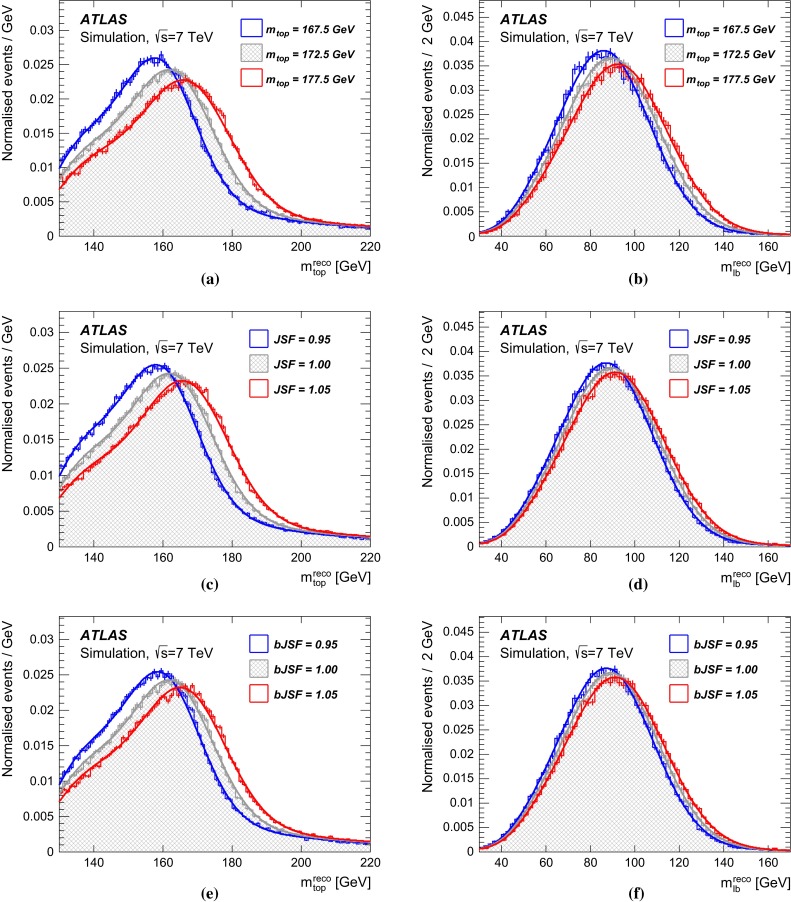
Distributions of $m_{\mathrm {top}} ^{\mathrm {reco}}$ in the $t\bar{t}\rightarrow \text{ lepton+jets }$ channel (*left*) and $m_{\ell b}^{\mathrm {reco}}$ in the $t\bar{t}\rightarrow \text{ dilepton }$ channel (*right*) and their template parameterisations for the signal, composed of simulated $t\bar{t}$ and single top quark production events. The expected sensitivities of $m_{\mathrm {top}} ^{\mathrm {reco}}$ and $m_{\ell b}^{\mathrm {reco}}$ are shown for events with at least two (or exactly two) $b\text{-tagged }$ jets. Figures **a** and **b** report the distributions for different values of the input $m_{\mathrm {top}}$ (167.5, 172.5 and 177.5 ${\mathrm { GeV}}$). Figures **c**, **d** and **e**, **f** show the $m_{\mathrm {top}} ^{\mathrm {reco}}$ and $m_{\ell b}^{\mathrm {reco}}$ distribution for $m_{\mathrm {top}}$ $=$ 172.5 ${\mathrm { GeV}}$, obtained with $\text{ JSF }$ or $\text{ bJSF }$ of 0.95, 1.00 and 1.05, respectively. Each distribution is overlaid with the corresponding probability density function that is obtained from the combined fit to all signal templates for all abservables

**Fig. 3:**
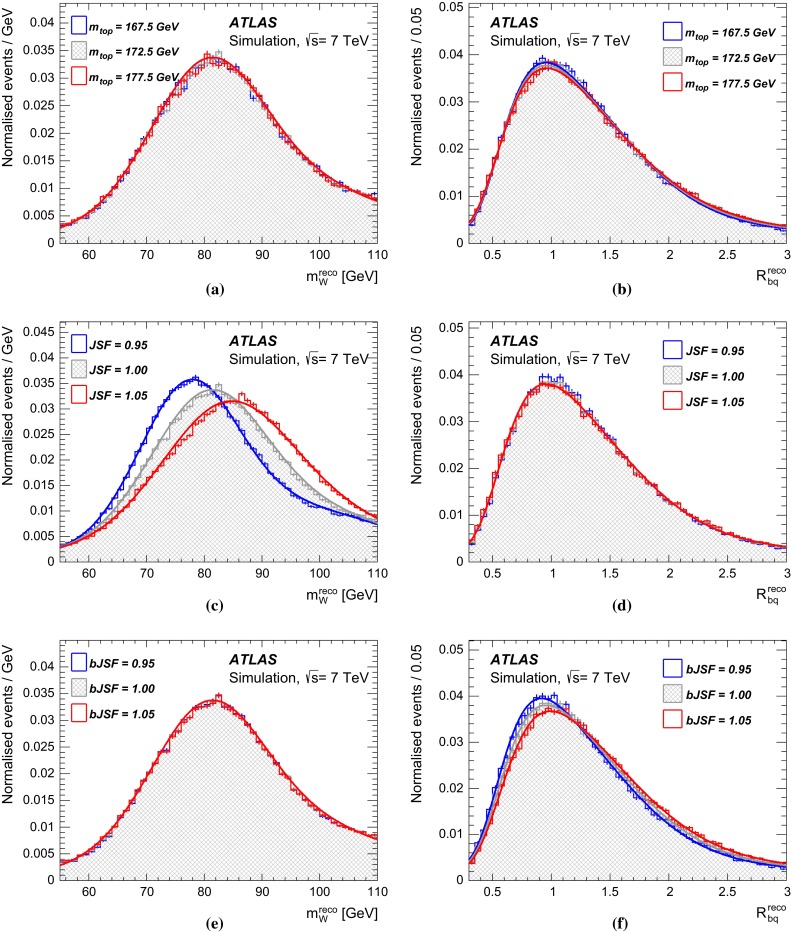
Distributions of $m_{W}^{\mathrm {reco}}$ (*left*) and $R_{b q} ^{\mathrm {reco}}$ (*right*) in the $t\bar{t}\rightarrow \text{ lepton+jets }$ channel and their template parameterisations for the signal, composed of simulated $t\bar{t}$ and single top quark production events. The expected sensitivity of $m_{W}^{\mathrm {reco}}$ and $R_{b q} ^{\mathrm {reco}}$ are shown for events with at least two $b\text{-tagged }$ jets. Figures **a** and **b** report the distributions for different values of the input $m_{\mathrm {top}}$ (167.5, 172.5 and 177.5 ${\mathrm { GeV}}$). Figures **c**, **d** and **e**, **f** show the $m_{W}^{\mathrm {reco}}$ and $R_{b q} ^{\mathrm {reco}}$ distribution for $m_{\mathrm {top}}$ $=$ 172.5 ${\mathrm { GeV}}$, obtained with $\text{ JSF }$ or $\text{ bJSF }$ of 0.95, 1.00 and 1.05, respectively. Each distribution is overlaid with the corresponding probability density function that is obtained from the combined fit to all signal templates for all abservables

The dependencies of the $m_{\mathrm {top}} ^{\mathrm {reco}}$ and $m_{\ell b}^{\mathrm {reco}}$ distributions on the underlying $m_{\mathrm {top}}$ used in the MC simulation are shown Fig. 2a and b, for events with at least (exactly) two $b\text{-tagged }$ jets, for the $t\bar{t}\rightarrow \text{ lepton+jets }$ ($t\bar{t}\rightarrow \text{ dilepton }$) channel. The $m_{\mathrm {top}} ^{\mathrm {reco}}$ and $m_{\ell b}^{\mathrm {reco}}$ distributions shown in Fig. 2c–f, exhibit sizeable sensitivity to global shifts of the $\text{ JSF }$ and the $\text{ bJSF }$. These effects introduce large systematic uncertainties on $m_{\mathrm {top}}$ originating from the uncertainties on the JES and bJES, unless additional information is exploited. As shown for the $t\bar{t}\rightarrow \text{ lepton+jets }$ channel in Fig. 3a, c and e, the $m_{W}^{\mathrm {reco}}$ distribution is sensitive to changes of the $\text{ JSF }$, while preserving its shape under variations of the input $m_{\mathrm {top}}$ and $\text{ bJSF }$. As originally proposed in Ref. [17], a simultaneous fit to $m_{\mathrm {top}} ^{\mathrm {reco}}$ and $m_{W}^{\mathrm {reco}}$ is used to mitigate the JES uncertainty. The $R_{b q} ^{\mathrm {reco}}$ distributions show substantial sensitivity to the $\text{ bJSF }$, and some dependence on the assumed $m_{\mathrm {top}}$ in the simulation, Fig. 3b, d and f. Complementing the information carried by the $m_{\mathrm {top}} ^{\mathrm {reco}}$ and $m_{W}^{\mathrm {reco}}$ observables, $R_{b q} ^{\mathrm {reco}}$ is used in an unbinned likelihood fit to the data to simultaneously determine $m_{\mathrm {top}}$, $\text{ JSF }$, and $\text{ bJSF }$. The per-event correlations of any pair of observables ($m_{\mathrm {top}} ^{\mathrm {reco}}$, $m_{W}^{\mathrm {reco}}$, and $R_{b q} ^{\mathrm {reco}}$) are found to be smaller than 0.15 and are neglected in this procedure.

### Templates and fits in the $t\bar{t}\rightarrow \text{ lepton+jets }$ channel

Signal templates are derived for the three observables for all $m_{\mathrm {top}}$-dependent samples, consisting of the $t\bar{t}$ signal events, together with single top quark production events. The signal templates for the $m_{\mathrm {top}} ^{\mathrm {reco}}$, $m_{W}^{\mathrm {reco}}$ and $R_{b q} ^{\mathrm {reco}}$ distributions are fitted to the sum of a Gaussian function and a Landau function for $m_{\mathrm {top}} ^{\mathrm {reco}}$ and $R_{b q} ^{\mathrm {reco}}$, and to a sum of two Gaussian functions for $m_{W}^{\mathrm {reco}}$ (Figs. 2, 3). For the background, the $m_{\mathrm {top}} ^{\mathrm {reco}}$ distribution is fitted to a Landau function, while both the $m_{W}^{\mathrm {reco}}$ and the $R_{b q} ^{\mathrm {reco}}$ distributions are fitted to the sum of two Gaussian functions. To exploit the different sensitivities to the underlying $m_{\mathrm {top}}$, $\text{ JSF }$ and $\text{ bJSF }$, all template fits are performed separately for events with one $b\text{-tagged }$ jet, and for events with at least two $b\text{-tagged }$ jets.

From individual fits to all signal templates listed above, it was verified that the parameters of the fitting functions depend linearly on the respective parameter $m_{\mathrm {top}}$, $\text{ JSF }$ or $\text{ bJSF }$. Consequently, this linearity is imposed when parametrising the fitting functions for the combined fit to all signal templates for the three observables. For the signal, the parameters of the fitting functions for $m_{\mathrm {top}} ^{\mathrm {reco}}$ depend linearly on $m_{\mathrm {top}}$, $\text{ JSF }$ and $\text{ bJSF }$. The parameters of the fitting functions of $m_{W}^{\mathrm {reco}}$ depend linearly on the $\text{ JSF }$. Finally, the parameters of the fitting functions of $R_{b q} ^{\mathrm {reco}}$ depend linearly on the $\text{ bJSF }$ and on $m_{\mathrm {top}}$. As shown in Fig. 3, the dependencies of $m_{W}^{\mathrm {reco}}$ on $m_{\mathrm {top}}$ and $\text{ bJSF }$, and of $R_{b q} ^{\mathrm {reco}}$ on $\text{ JSF }$ are negligible. For the background, the parameter dependencies of the fitting functions are the same except that, by construction, they do not depend on $m_{\mathrm {top}}$.

Signal and background probability density functions $P^{\mathrm {sig}}$ and $P^{\mathrm {bkg}}$ for the $m_{\mathrm {top}} ^{\mathrm {reco}}$, $m_{W}^{\mathrm {reco}}$ and $R_{b q} ^{\mathrm {reco}}$ distributions are used in an unbinned likelihood fit to the data for all events, $i=1,\dots N$. The likelihood function maximised is:

1 $$\begin{aligned}&\mathcal{L}_{\mathrm{shape}}^{\ell \mathrm{+jets}} (m_{\mathrm {top}}, \text{ JSF }, \text{ bJSF }, f_{\mathrm {bkg}})\\& = \prod _{i=1}^{N} P_{\mathrm {top}} (m_{\mathrm {top}} ^{\mathrm {reco},i} \,\vert \,m_{\mathrm {top}}, \text{ JSF }, \text{ bJSF }, f_{\mathrm {bkg}}) \nonumber \\& \times \,P_{W} (m_{W}^{\mathrm {reco},i} \,\vert \,\text{ JSF }, f_{\mathrm {bkg}}) \\& \times\, P_{bq} (R_{b q} ^{\mathrm {reco},i} \,\vert \,m_{\mathrm {top}},\text{ bJSF }, f_{\mathrm {bkg}}), \end{aligned}$$

with:

$$\begin{aligned}&P_{\mathrm {top}} (m_{\mathrm {top}} ^{\mathrm {reco},i} \,\vert \,m_{\mathrm {top}}, \text{ JSF }, \text{ bJSF }, f_{\mathrm {bkg}}) \\& = (1-f_{\mathrm {bkg}})\cdot P_{\mathrm {top}}^{\mathrm {sig}} (m_{\mathrm {top}} ^{\mathrm {reco},i} \,\vert \,m_{\mathrm {top}}, \text{ JSF }, \text{ bJSF }) \\&\quad +\, f_{\mathrm {bkg}} \cdot P_{\mathrm {top}}^{\mathrm {bkg}} (m_{\mathrm {top}} ^{\mathrm {reco},i} \,\vert \,\text{ JSF }, \text{ bJSF }), \\&P_{W} (m_{W}^{\mathrm {reco},i} \,\vert \,\text{ JSF }, f_{\mathrm {bkg}}) \\& = (1-f_{\mathrm {bkg}})\cdot P_{W}^{\mathrm {sig}} (m_{W}^{\mathrm {reco},i} \,\vert \,\text{ JSF }) \\&\quad +\, f_{\mathrm {bkg}} \cdot P_{W}^{\mathrm {bkg}} (m_{W}^{\mathrm {reco},i} \,\vert \,\text{ JSF }), \\&P_{bq} (R_{b q} ^{\mathrm {reco},i} \,\vert \,m_{\mathrm {top}},\text{ bJSF }, f_{\mathrm {bkg}})\\& = (1-f_{\mathrm {bkg}})\cdot P_{bq}^{\mathrm {sig}} (R_{b q} ^{\mathrm {reco},i} \,\vert \,m_{\mathrm {top}},\text{ bJSF }) \\&\quad +\, f_{\mathrm {bkg}} \cdot P_{bq}^{\mathrm {bkg}} (R_{b q} ^{\mathrm {reco},i} \,\vert \,\text{ bJSF }) \end{aligned}$$

where the fraction of background events is denoted by $f_{\mathrm {bkg}}$. The parameters to be determined by the fit are $m_{\mathrm {top}}$, $\text{ JSF }$, $\text{ bJSF }$ and $f_{\mathrm {bkg}}$, where $f_{\mathrm {bkg}}$ is determined separately for the $t\bar{t}\rightarrow \text{ lepton+jets }$ data sets with exactly one or at least two $b\text{-tagged }$ jets.

Pseudo-experiments are used to verify the internal consistency of the fitting procedure and to obtain the expected statistical uncertainty corresponding to a data sample of $$4.6 $$ $$\text{ fb }^{-1}$$. For each choice of the input parameters, 500 pseudo-experiments are generated. To retain the correlation of the analysis observables, individual MC events drawn from the full simulated event samples are used, rather than sampling from the separate $$m_{\mathrm {top}} ^{\mathrm {reco}} $$, $$m_{W}^{\mathrm {reco}} $$, and $$R_{b q} ^{\mathrm {reco}} $$ distributions. For all five parameters, good linearity is found between the input parameters used to perform the pseudo-experiments, and the results of the fits. Within their statistical uncertainties, the mean values and widths of the pull distributions are consistent with the expectations of zero and one, respectively. This means the method is unbiased with appropriate statistical uncertainties. The expected statistical uncertainties on $$m_{\mathrm {top}} $$ including the statistical contributions from the simultaneous fit of the $$\text{ JSF } $$ and $$\text{ bJSF } $$ obtained from pseudo-experiments at an input top quark mass of $$m_{\mathrm {top}} =172.5~ {\mathrm { GeV}}$$, and for a luminosity of $$4.6 \text{ fb }^{-1}$$, are $$1.50 \pm 0.06~ {\mathrm { GeV}}$$ and $$0.89 \pm 0.01~ {\mathrm { GeV}}$$ for the case of one $$b\text{-tagged } $$ jet and for the case of at least two $$b\text{-tagged } $$ jets, respectively. The results correspond to the mean value and the standard deviation of the distribution of the statistical uncertainties of the fitted masses from the pseudo-experiments. The different expected statistical uncertainties on $$m_{\mathrm {top}} $$ for the samples with different numbers of $$b\text{-tagged } $$ jets, which are obtained from samples containing similar numbers of events (see Table 1), are mainly a consequence of the different resolution on $$m_{\mathrm {top}} $$.

### Templates and fits in the $t\bar{t}\rightarrow \text{ dilepton }$ channel

The signal $m_{\ell b}^{\mathrm {reco}}$ templates comprise both the $t\bar{t}$ and the single top quark production processes, and are fitted to the sum of a Gaussian function and a Landau function, while the background distribution is fitted to a Landau function. Similarly to the $t\bar{t}\rightarrow \text{ lepton+jets }$ channel, all template fits are performed separately for events with one $b\text{-tagged }$ jet, and for events with exactly two $b\text{-tagged }$ jets. In Fig. 2b the sensitivity of the $m_{\ell b}^{\mathrm {reco}}$ observable to the input value of the top quark mass is shown for the events with exactly two $b\text{-tagged }$ jets, by the superposition of the signal templates and their fits for three input $m_{\mathrm {top}}$ values. For the signal templates, the parameters of the fitting functions of $m_{\ell b}^{\mathrm {reco}}$ depend linearly on $m_{\mathrm {top}}$.

Signal and background probability density functions for the $m_{\ell b}^{\mathrm {reco}}$ estimator are built, and used in an unbinned likelihood fit to the data for all events, $i=1,\dots N$. The likelihood function maximised is:

2 $$\begin{aligned}&{\mathcal {L}_{\mathrm {shape}}^{\mathrm {dilepton}}} (m_{\mathrm {top}}, f_{\mathrm {bkg}})\nonumber \\&\quad = \prod _{i=1}^{N} [ (1-f_{\mathrm {bkg}})\cdot P_{\mathrm {top}}^{\mathrm {sig}} ({m_{\ell b}^{\mathrm {reco},i}} \,\vert \, m_{\mathrm {top}}) + f_{\mathrm {bkg}} \cdot P_{\mathrm {top}}^{\mathrm {bkg}} ({m_{\ell b}^{\mathrm {reco},i}}) ], \end{aligned}$$

where, as for the $t\bar{t}\rightarrow \text{ lepton+jets }$ case, $P_{\mathrm {top}}^{\mathrm {sig}}$ and $P_{\mathrm {top}}^{\mathrm {bkg}}$ are the signal and background probability density functions and $f_{\mathrm {bkg}}$ is the fraction of background events in the selected data set.

Using pseudo-experiments, also for this decay channel good linearity is found between the input top quark mass used to perform the pseudo-experiments, and the results of the fits. Within their statistical uncertainties, the mean values and widths of the pull distributions are consistent with the expectations of zero and one, respectively. The expected statistical uncertainties on $$m_{\mathrm {top}} $$ obtained from pseudo-experiments for an input top quark mass of $$m_{\mathrm {top}} =172.5$$ $${\mathrm { GeV}}$$, and for a luminosity of $$4.6 $$ $$\text{ fb }^{-1}$$, are $$0.95 \pm 0.04~ {\mathrm { GeV}}$$ and $$0.65\pm 0.02 ~ {\mathrm { GeV}}$$ for events with exactly one or two $$b\text{-tagged } $$ jets, respectively. As for the $$\ell \text{+jets } $$ channel, the different expected statistical uncertainties on $$m_{\mathrm {top}} $$ for the samples with different numbers of $$b\text{-tagged } $$ jets, which are obtained from samples containing similar numbers of events (see Table 1), are mainly a consequence of the different resolution on $$m_{\mathrm {top}} $$.

### Combined likelihood fit to the event samples

The final results for both the $\ell \text{+jets }$ and $\text{ dilepton }$ final states are obtained combining at the likelihood level the events with one or more $b\text{-tagged }$ jets. The measured $m_{\mathrm {top}}$ is assumed to be the same in these two sub-samples per decay channel. Similarly, the $\text{ JSF }$ and the $\text{ bJSF }$ are taken to be the same for the samples of the $t\bar{t}\rightarrow \text{ lepton+jets }$ analysis with different $b\text{-tagged }$ jet multiplicities. On the contrary, the background fractions for the two decay channels, and for the samples with different numbers of $b\text{-tagged }$ jets, are kept independent, corresponding to four individual parameters ($f_\mathrm{bkg}^{\ell +\mathrm{jets}, 1b}$, $f_\mathrm{bkg}^{\ell +\mathrm{jets}, 2b}$, $f_\mathrm{bkg}^{\mathrm{dil}, 1b}$, $f_\mathrm{bkg}^{\mathrm{dil}, 2b}$).

The combined likelihood fit allows the statistical uncertainties on the fitted parameters to be reduced, while mitigating some systematic effects. The expected statistical precision on $$m_{\mathrm {top}} $$, for an input top quark mass of $$m_{\mathrm {top}} =172.5$$ $${\mathrm { GeV}}$$, a luminosity of $$4.6 $$ $$\text{ fb }^{-1}$$, and in the combined one or more $$b\text{-tagged } $$ jets event sample, is $$0.76 \pm 0.01~ {\mathrm { GeV}}$$ and $$0.54 \pm 0.01~ {\mathrm { GeV}}$$ for the $$t\bar{t}\rightarrow \text{ lepton+jets } $$ and $$t\bar{t}\rightarrow \text{ dilepton } $$ analyses, respectively.

## Top quark mass measurements

The results of the fits for the $t\bar{t}\rightarrow \text{ lepton+jets }$ and $t\bar{t}\rightarrow \text{ dilepton }$ analyses are:

$$\begin{aligned} {m_{\mathrm {top}}^{\ell \mathrm {+jets}}}= & {} {172.33} \pm {0.75} ~\mathrm {(stat + \text{ JSF } + \text{ bJSF })}~{\mathrm { GeV}},\\ \text{ JSF }= & {} {{1.019} \,\pm {0.003} ~\mathrm {(stat)}},\\ \text{ bJSF }= & {} {{1.003} \,\pm {0.008} ~\mathrm {(stat)}}, \\ {m_{\mathrm {top}}^{\mathrm {dil}}}= & {} {{173.79} \,\pm {0.54} ~\mathrm {(stat)}} ~{\mathrm { GeV}}. \end{aligned}$$

For the $t\bar{t}\rightarrow \text{ lepton+jets }$ channel, the fitted background fractions amount to $18.4 \pm 2.2\,\%$ and $2.4\pm 1.5\,\%$ for one $b\text{-tagged }$ jet and the at least two $b\text{-tagged }$ jets samples respectively. The corresponding values for the $t\bar{t}\rightarrow \text{ dilepton }$ analysis are $3.5 \pm 3.7\,\%$ and $1.4\pm 2.2\,\%$ for one $b\text{-tagged }$ jet and the two $b\text{-tagged }$ jets samples respectively. All quoted uncertainties are statistical only. These fractions are consistent with the expectations given in Table 1. The correlation matrices for the fitted parameters in the $t\bar{t}\rightarrow \text{ lepton+jets }$ and $t\bar{t}\rightarrow \text{ dilepton }$ analyses are reported in Table 2.

**Table 2:** The correlations of the fitted parameters used in the likelihood maximisation of the $t\bar{t}\rightarrow \text{ lepton+jets }$ analysis (top) and the $t\bar{t}\rightarrow \text{ dilepton }$ analysis (bottom)

	${m_{\mathrm {top}}^{\ell \mathrm {+jets}}}$	$\text{ JSF }$	$\text{ bJSF }$	$f_\mathrm{bkg}^{\ell +\mathrm{jets}, 1b}$	$f_\mathrm{bkg}^{\ell +\mathrm{jets}, 2b}$
${m_{\mathrm {top}}^{\ell \mathrm {+jets}}}$	1.00				
$\text{ JSF }$	$-0.36$	1.00			
$\text{ bJSF }$	$-0.89$	0.03	1.00		
$f_\mathrm{bkg}^{\ell +\mathrm{jets}, 1b}$	$-0.03$	$-0.01$	0.06	1.00	
$f_\mathrm{bkg}^{\ell +\mathrm{jets}, 2b}$	$-0.06$	$-0.09$	0.09	0.01	1.00

	${m_{\mathrm {top}}^{\mathrm {dil}}}$	$f_\mathrm{bkg}^{\mathrm{dil}, 1b}$	$f_\mathrm{bkg}^{\mathrm{dil}, 2b}$		
${m_{\mathrm {top}}^{\mathrm {dil}}}$	1.00				
$f_\mathrm{bkg}^{\mathrm{dil}, 1b}$	0.07	1.00			
$f_\mathrm{bkg}^{\mathrm{dil}, 2b}$	$-0.14$	$-0.01$	1.00		

**Fig. 4:**
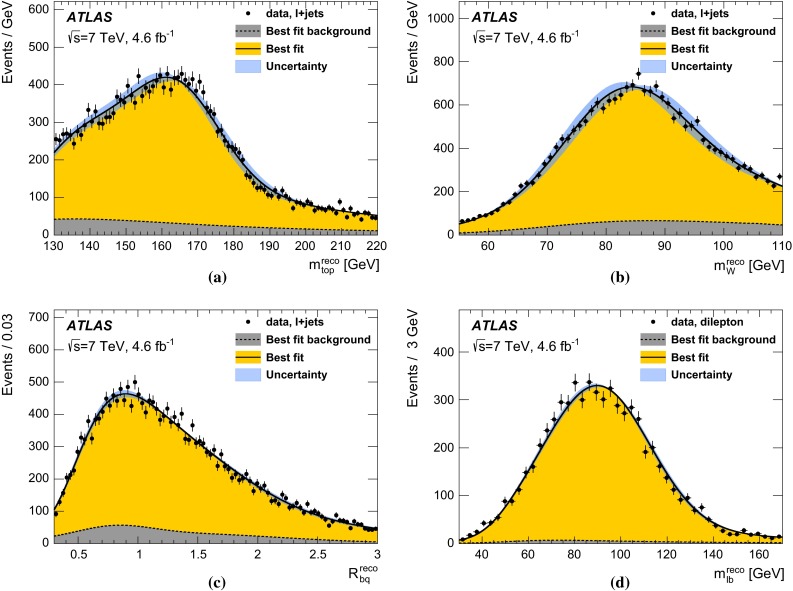
The fitted distributions in the data, showing **a** $m_{\mathrm {top}} ^{\mathrm {reco}}$, **b** $m_{W}^{\mathrm {reco}}$, **c** $R_{b q} ^{\mathrm {reco}}$, and **d** $m_{\ell b}^{\mathrm {reco}}$. The fitted probability density functions for the background alone and for signal-plus-background are also shown. The uncertainty bands indicate the total uncertainty on the signal-plus-background fit obtained from pseudo-experiments as explained in the text. Figures **a**–**c** refer to the $t\bar{t}\rightarrow \text{ lepton+jets }$ analysis, figure **d** to the $t\bar{t}\rightarrow \text{ dilepton }$ analysis

Figure 4 shows the $m_{\mathrm {top}} ^{\mathrm {reco}}$, $m_{W}^{\mathrm {reco}}$, $R_{b q} ^{\mathrm {reco}}$ and $m_{\ell b}^{\mathrm {reco}}$ distributions in the data together with the corresponding fitted probability density functions for the background alone and for the sum of signal and background. The uncertainty bands are obtained by varying the three fitted parameters $m_{\mathrm {top}}$, $\text{ JSF }$, and $\text{ bJSF }$ within $\pm 1\sigma$ of their full uncertainties taking into account their correlation, while keeping the background fractions fixed. The individual systematic uncertainties and the correlations are discussed in Sects. 7 and  8, respectively. The band shown is the envelope of all probability density functions obtained from 500 pseudo-experiments varying the parameters. Within this band, the data are well described by the fitted probability density function.

**Fig. 5:**
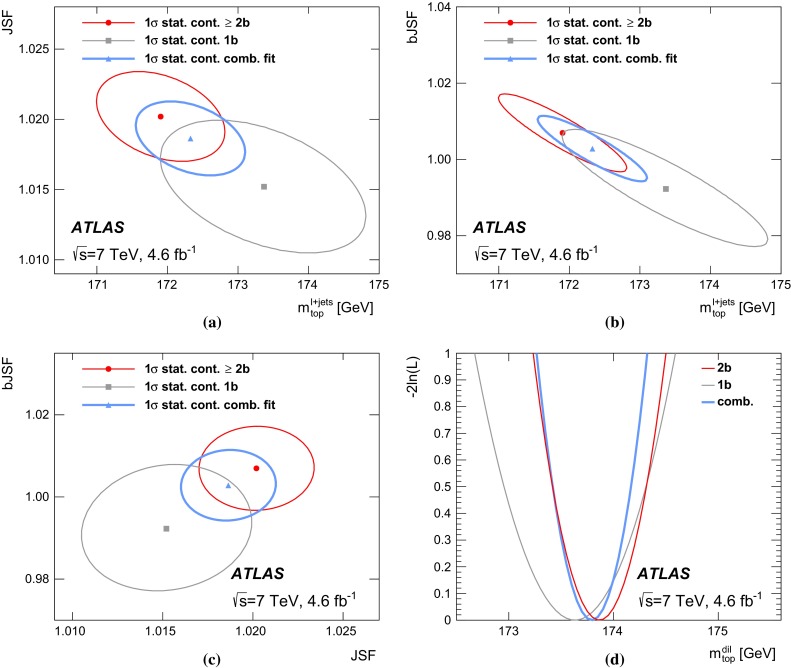
Likelihood contours showing the correlation determined in data of the measured ${m_{\mathrm {top}}^{\ell \mathrm {+jets}}}$ to **a** the $\text{ JSF }$ and **b** the $\text{ bJSF }$, and **c** the correlation of the two scales $\text{ JSF }$ and $\text{ bJSF }$, within the $t\bar{t}\rightarrow \text{ lepton+jets }$ analysis. Figures **a**–**c** show the results using the events with one $b\text{-tagged }$ jet only (*grey ellipses*), with at least two $b\text{-tagged }$ jets (*red ellipses*) and finally with all selected events, i.e. the ones with at least one $b\text{-tagged }$ jet (*blue ellipses*). The *ellipses* correspond to the $\pm 1\sigma$ (statistical) uncertainties, including the statistical components from the $\text{ JSF }$ and $\text{ bJSF }$ determination. While tracing the contours the additional parameters of the likelihood are fixed to their best fit values. Figure **d** reports the likelihood profile as a function of ${m_{\mathrm {top}}^{\mathrm {dil}}}$ for the sample with one $b\text{-tagged }$ jet, the sample with two $b\text{-tagged }$ jets and the combined result. The *colour coding* is analogous to figures **a**–**c**

For the $t\bar{t}\rightarrow \text{ lepton+jets }$ analysis, the measured values of the three observables (${m_{\mathrm {top}}^{\ell \mathrm {+jets}}}$, $\text{ JSF }$, and $\text{ bJSF }$), together with two-dimensional statistical uncertainty contours ($\pm 1\sigma$), including the statistical components from the $\text{ JSF }$ and $\text{ bJSF }$ determination, are shown in Fig. 5a–c. Correspondingly, the likelihood profile as a function of ${m_{\mathrm {top}}^{\mathrm {dil}}}$ is reported in Fig. 5d, for the sample with one $b\text{-tagged }$ jet, the sample with two $b\text{-tagged }$ jets and the combined $t\bar{t}\rightarrow \text{ dilepton }$ result. These results demonstrate the good agreement between the parameter values measured in the samples with different $b\text{-tagged }$ jet multiplicities.

## Uncertainties affecting the $m_{\mathrm {top}}$ determination

Several sources of systematic uncertainty are considered. Their effects on the $\ell \text{+jets }$ and $\text{ dilepton }$ measurements are listed in Table 3, together with the result of the combination of the two channels discussed in Sect. 8. Each source of uncertainty considered is investigated, when possible, by varying the relevant quantities by $\pm 1\sigma$ with respect to their default values. Using the changed parameters, 500 pseudo-experiments are performed using events drawn from the full simulated samples. The difference of the average $m_{\mathrm {top}}$ computed from pseudo-experiments based on the standard MC sample, and the varied sample under consideration, both evaluated with the original template parameterisations, is used to determine the corresponding uncertainty. Unless stated otherwise, the systematic uncertainties arising from the different modelling sources are calculated as half of the difference of the results of the upward and downward variations. The systematic uncertainties for the measured $\text{ JSF }$ and $\text{ bJSF }$ in the $t\bar{t}\rightarrow \text{ lepton+jets }$ final state are also estimated. Following Ref. [67], the actual observed difference is quoted as the systematic uncertainty on the corresponding source, even if it is smaller than its associated statistical precision. The latter is estimated taking into account the statistical correlation of the MC samples used in the comparison. The total uncertainty is calculated as the sum in quadrature of all individual contributions, i.e. neglecting possible correlations (small by construction). The estimation of the uncertainties for the individual contributions is described in the following.

### Statistics and method calibration

#### Statistical components due to the jet energy scale factors

The statistical uncertainty quoted for the $t\bar{t}\rightarrow \text{ lepton+jets }$ analysis is made up of three parts: a purely statistical component on $m_{\mathrm {top}}$ and the contributions stemming from the simultaneous determination of the $\text{ JSF }$ and $\text{ bJSF }$. The former is obtained from a one-dimensional template method exploiting only the $m_{\mathrm {top}} ^{\mathrm {reco}}$ observable (fixing the values of the $\text{ JSF }$ and $\text{ bJSF }$ to the results of the three-dimensional analysis). The contribution to the statistical uncertainty on the fitted parameters due to the simultaneous fit of $m_{\mathrm {top}}$ and $\text{ JSF }$, is estimated as the difference in quadrature of the statistical uncertainty of a two-dimensional ($m_{\mathrm {top}} ^{\mathrm {reco}}$ and $m_{W}^{\mathrm {reco}}$, fixing the value of $\text{ bJSF }$) fit and the one-dimensional fit to the data described above. Analogously, the contribution of the statistical uncertainty due to the simultaneous fit of $\text{ bJSF }$ together with $m_{\mathrm {top}}$ and $\text{ JSF }$, is defined as the difference in quadrature of the statistical uncertainties obtained in the three-dimensional and the two-dimensional (fixing $\text{ bJSF }$) fits to the data. This separation allows a direct comparison of the sensitivity of the $m_{\mathrm {top}}$ estimator for any analysis, irrespective of the number of observables exploited by the fit. In addition, the sensitivity of the estimators for the global jet energy scales can be directly compared. These uncertainties can be treated as uncorrelated uncertainties in $m_{\mathrm {top}}$ combinations. Together with the systematic components of the residual jet energy scale uncertainty discussed in Sect. 7.4 below, they directly replace the uncertainty on $m_{\mathrm {top}}$ from the jet energy scale variations present without the in situ determination.

#### Method calibration

This uncertainty takes into account the effect of any bias introduced in the fit by the presence of correlations among the observables (neglected in the fit for the $t\bar{t}\rightarrow \text{ lepton+jets }$ analysis) as well as the impact of the limited size of the MC samples (for both analyses). This leads to a systematic uncertainty in the template fit, which is reflected in the residual mass differences of the fitted mass and the input mass for a given MC sample. The largest average difference observed in the pseudo-experiments carried out varying the underlying top quark mass, the $\text{ JSF }$ and the $\text{ bJSF }$ with respect to the respective input parameter, is taken as the uncertainty from this source.

### $t\bar{t}$ modelling

#### Signal Monte Carlo generator

The systematic uncertainty related to the choice of $t\bar{t}$ signal generator program is determined by comparing the results of pseudo-experiments performed with either the MC@NLO  [68, 69] samples or the Powheg samples, both generated with $m_{\mathrm {top}} =172.5$ ${\mathrm { GeV}}$ and using the Herwig program to perform the hadronisation. This choice is supported by the observation that these MC@NLO and Powheg samples exhibit very different jet multiplicities for the $t\bar{t}\rightarrow \text{ lepton+jets }$ channel which bracket those observed in data [70]. The full difference of the results averaged over all pseudo experiments is quoted as the systematic uncertainty.

The impact of changing the factorisation and renormalisation scales ($\mu _{\mathrm {F/R}}$) in Powheg was also checked. The resulting $m_{\mathrm {top}}$ systematic uncertainties amount to $0.15 \pm 0.07~{\mathrm { GeV}}$ and $0.14\pm 0.05~{\mathrm { GeV}}$ for the $t\bar{t}\rightarrow \text{ lepton+jets }$ channel, and $t\bar{t}\rightarrow \text{ dilepton }$ analysis respectively. Within the quoted statistical uncertainties, the $\mu _{\mathrm { F/R}}$ systematic uncertainties are consistent with those originating from the comparison of MC@NLO and Powheg, which are used here.

#### Hadronisation

Signal samples for $m_{\mathrm {top}} =172.5$ ${\mathrm { GeV}}$ from the Powheg event generator are produced performing the parton showering and the hadronisation with either Pythia with the P2011C tune or Herwig and Jimmy with the ATLAS AUET2 tune [50]. The full difference of the results averaged over all pseudo experiments is quoted as the systematic uncertainty.

#### Initial- and final-state QCD radiation

Different amounts of initial- and final-state QCD radiation can alter the jet energies and multiplicities of the events, introducing distortions into the measured $m_{\mathrm {top}} ^{\mathrm {reco}}$, $m_{W}^{\mathrm {reco}}$, $R_{b q} ^{\mathrm {reco}}$ and $m_{\ell b}^{\mathrm {reco}}$ distributions. This effect is evaluated by performing pseudo-experiments using two dedicated signal samples generated with AcerMC  [30] in combination with Pythia P2011C for hadronisation and parton showering. In these samples some Pythia P2011C parameters that control the showering are varied in ranges that are compatible with a study of additional jets in $t\bar{t}$ events [71], and half the difference of these two extremes is used as the systematic uncertainty.

#### Underlying event and colour reconnection

These systematic uncertainties are estimated using samples simulated with Powheg-hvq and Pythia. The underlying-event uncertainty is obtained by comparing a sample with the Perugia 2012 tune (P2012) to a sample with the P2012 mpiHi tune [28]. The full difference in the fitted mass of the two models is taken as the systematic uncertainty for this source. Similarly, the colour reconnection systematic uncertainty is assigned as the difference in the fitted parameters of samples obtained with the P2012 and P2012 loCR tunes [28]. The same matrix-element-level Powheg-hvq events generated with the CT10 PDFs are used for the three MC samples. The P2012 mpiHi tune is a variation of the P2012 tune with more semi-hard multiple parton interactions. The colour reconnection parameters were kept fixed to the P2012 tune values. Compared to the standard P2012 tune the P2012 loCR tune leads to significantly less activity in the transverse region with respect to the leading charged-particle as measured in Ref. [51]. In addition to assessing the effect of colour reconnection, this tune is therefore also used to estimate the systematic uncertainty associated with the particle spectra in the underlying event.

#### Parton distribution functions

The signal samples are generated using the CT10 PDFs. These PDFs, obtained from experimental data, have an uncertainty that is reflected in 26 pairs of possible PDF variations provided by the CTEQ group. To evaluate the impact of the PDF uncertainty on the $t\bar{t}$ signal templates, the events, from a sample generated using MC@NLO with Herwig fragmentation, are re-weighted with the corresponding ratio of PDFs, and 26 pairs of signal templates are constructed, one pair per PDF uncertainty. For each pair, the average measured $m_{\mathrm {top}}$ is obtained from 500 pseudo-experiments each for the upward and downward variations of the PDF uncertainty. The corresponding uncertainty is obtained as half the difference of the two values. From those the CT10 contribution is calculated as the sum in quadrature of the 26 uncertainties and amounts to 0.13 ${\mathrm { GeV}}$ and 0.10 ${\mathrm { GeV}}$ for the $t\bar{t}\rightarrow \text{ lepton+jets }$ and $t\bar{t}\rightarrow \text{ dilepton }$ analysis respectively.

In addition, the signal $t\bar{t}$ samples are re-weighted to match the central PDFs for either the MSTW2008 [38] or the NNPDF23 [41] PDFs. The corresponding differences, taken as uncertainties, are 0.03 ${\mathrm { GeV}}$ and 0.21 ${\mathrm { GeV}}$ for the $t\bar{t}\rightarrow \text{ lepton+jets }$ analysis, and 0.01 ${\mathrm { GeV}}$ and 0.01 ${\mathrm { GeV}}$ for the $t\bar{t}\rightarrow \text{ dilepton }$ analysis. The final PDF systematic uncertainty is the sum in quadrature of the three contributions discussed above.

### Modelling of non-$t\bar{t}$ processes

The uncertainty in the modelling of non-$t\bar{t}$ processes is taken into account by varying the normalisation and the shape of the distributions of several contributions.

The uncertainty on the $W \text{+jets }$ background determined from data [64] is dominated by the uncertainty on the heavy-flavour content of these events and amounts to ${\pm 30~\%}$ of the overall normalisation. The same normalisation uncertainty is assigned to the $Z \text{+jets }$ background normalisation. Uncertainties related to the $W \text{+jets }$ background shape are also considered. These stem from the variation of the heavy-flavour composition of the samples and from re-weightings of the distributions to match the predictions of Alpgen. For the re-weighting, parameters are varied which affect the functional form of the factorisation and renormalisation scales, and the threshold for the matching scale used to connect the matrix-element calculation to the parton shower.

The estimate of the background from NP/fake leptons determined from data is varied by ${\pm 50~\%}$ to account for the uncertainty of this background source [65]. Uncertainties affecting the shape of this background are also included. For the NP/fake-electron background, the effects on the shape arising from the efficiency uncertainties for real and fake electrons are evaluated and added in quadrature. For the NP/fake-muon background, two different matrix methods were used and averaged: their difference is taken as the systematic uncertainty.

In addition, the impact of changing the normalisation of the single top quark processes according to the uncertainty on the corresponding theoretical cross sections is considered. This yields a negligible systematic uncertainty in both the $t\bar{t}\rightarrow \text{ lepton+jets }$ and $t\bar{t}\rightarrow \text{ dilepton }$ analyses.

### Detector modelling

#### Jet energy scale

The JES is derived using information from test-beam data, LHC collision data, and simulation. The relative JES uncertainty varies from about 1 % to 3 % depending on jet $p_{\text {T}}$ and $\eta$ as given in Ref. [58]. Since the estimation of the jet energy scale involves a number of steps, the JES uncertainty has various components originating from the calibration method, the calorimeter response, the detector simulation, and the specific choice of parameters in the physics model employed in the MC event generator. The total uncertainty is expressed in terms of 21 $p_{\text {T}}$- and $\eta$-dependent components which are considered uncorrelated [58]. The uncertainties for the individual components and their sum are given in Table 4 in Appendix A. Despite the simultaneous fit of $m_{\mathrm {top}}$, $\text{ JSF }$ and $\text{ bJSF }$ in the $t\bar{t}\rightarrow \text{ lepton+jets }$ channel there is a non-negligible residual JES uncertainty. This is introduced by the variation of the jet energy scale corrections and their uncertainties with jet kinematics, which cannot be fully captured by global scale factors ($\text{ JSF }$, $\text{ bJSF }$). However the overall JES uncertainty is a factor of two smaller than in a one-dimensional analysis exploiting only templates of $m_{\mathrm {top}} ^{\mathrm {reco}}$. In the $t\bar{t}\rightarrow \text{ dilepton }$ channel, the contribution from the JES uncertainty constitutes the main component of systematic uncertainty on $m_{\mathrm {top}}$.

#### b-Jet energy scale

This uncertainty is uncorrelated with the JES uncertainty and accounts for the remaining differences of $b\text{-jets }$ and light-jets after the global JES was determined. For this, an extra uncertainty ranging from 0.7 % to 1.8 % and depending on jet $p_{\text {T}}$ and $\eta$ is assigned to $b\text{-jets }$, due to differences between jets containing $b\hbox {-hadrons}$ and the inclusive jet sample [58]. This additional systematic uncertainty was obtained from MC simulation and was verified using $b\text{-tagged }$ jets in data. The validation of the $b\text{-jet }$ energy scale uncertainty is based on the comparison of the jet transverse momentum as measured in the calorimeter to the total transverse momentum of charged-particles associated with the jet. These transverse momenta are evaluated in the data and in MC simulated events for all jets and for $b\text{-jets }$ [58]. In addition, a validation using $t\bar{t}\rightarrow \text{ lepton+jets }$ events was performed. Effects stemming from $b\text{-quark }$ fragmentation, hadronisation and underlying soft radiation were studied using different MC event generation models [58]. Thanks to the simultaneous fit to $R_{b q} ^{\mathrm {reco}}$ together with $m_{W}^{\mathrm {reco}}$ and $m_{\mathrm {top}} ^{\mathrm {reco}}$, the $t\bar{t}\rightarrow \text{ lepton+jets }$ three-dimensional analysis method mitigates the impact of this uncertainty, and reduces it to 0.06 ${\mathrm { GeV}}$, instead of 0.88 ${\mathrm { GeV}}$ in a two-dimensional analysis method (exploiting two-dimensional templates of $m_{\mathrm {top}} ^{\mathrm {reco}}$ and $m_{W}^{\mathrm {reco}}$, as in Ref. [8]), albeit at the cost of an additional statistical component of 0.67 ${\mathrm { GeV}}$. In the $t\bar{t}\rightarrow \text{ dilepton }$ channel, the contribution from the bJES uncertainty represents the second largest component of systematic uncertainty on $m_{\mathrm {top}}$.

#### Jet energy resolution

To assess the impact of this uncertainty, before performing the event selection, the energy of each reconstructed jet in the simulation is smeared by a Gaussian function such that the width of the resulting Gaussian distribution corresponds to the one including the uncertainty on the jet energy resolution [72]. The fit is performed using smeared jets and the deviation from the central result is assigned as a systematic uncertainty.

#### Jet reconstruction efficiency

The jet reconstruction efficiency for data and the MC simulation is found to be in agreement with an accuracy of better than $\pm 2~\%$  [73]. To account for the residual uncertainties, 2 % of jets with $p_{\text {T}} < 30$ ${\mathrm { GeV}}$ are randomly removed from MC simulated events. The event selection and the fit are repeated on the changed sample. The changes in the fitted parameters relative to the nominal MC sample are assigned as systematic uncertainty.

#### Jet vertex fraction

Residual differences between data and MC in the description of the fraction of the jet momentum associated with tracks from the primary vertex (used to suppress pile-up interactions) is corrected by applying scale factors. These scale factors, varied according to their uncertainty, are applied to MC simulation events as a function of the jet $p_{\text {T}}$. The resulting variation in the measured top quark mass in the $t\bar{t}\rightarrow \text{ lepton+jets }$ analysis is 10  MeV, while it is negligible for the $t\bar{t}\rightarrow \text{ dilepton }$ analysis.

#### *b*-Tagging efficiency and mistag rate

To account for potential mismodelling of the $$b\text{-tagging } $$ efficiency and the mistag rate, $$b\text{-tagging } $$ scale factors, together with their uncertainties, are derived per jet [61–63, 74]. They are applied to the MC events and depend on the jet $$p_{\text {T}} $$ and $$\eta $$ and the underlying quark flavour. In this analysis these correction factors are obtained from dijet [62] and $$t\bar{t}\rightarrow \text{ dilepton } $$ events. The same $$b\text{-tagging } $$ calibrations are applied to both the $$\ell \text{+jets } $$ and $$\text{ dilepton } $$ final states. The $$t\bar{t}$$-based calibrations are obtained using the methodology described in Ref. [63], applied to the 7 $${\mathrm { TeV}}$$ data. The statistical correlation stemming from the use of partially overlapping data sets for the $$t\bar{t}\rightarrow \text{ dilepton } $$$$m_{\mathrm {top}} $$ analysis and the $$b\text{-tagging } $$ calibration is estimated to be negligible. The correlation of those systematic uncertainties that are in common for the $$b\text{-tagging } $$ calibration and the present analyses is taken into account. Similarly to the JES uncertainty, the uncertainty on the correction factors for the $$b\text{-tagging } $$ efficiency is separated into ten uncorrelated components. The systematic uncertainty is assessed by changing the correction factor central values by $$\pm 1\sigma $$ for each component, and performing the fit. The final uncertainty due to the $$b\text{-tagging } $$ efficiency is calculated as the sum in quadrature of all contributions. A similar procedure is applied for the mistag rates for $$ c \text{-jets }$$, albeit using four separate components. In addition, the correction factors and mistag rates for light-jets are varied within their uncertainty, and the corresponding shifts in the measured quantities are summed in quadrature. The size of the $$b\text{-tagging } $$ systematic uncertainty of 0.50 $${\mathrm { GeV}}$$ observed in the $$t\bar{t}\rightarrow \text{ lepton+jets } $$ analysis is mostly driven by the induced change in shape of the $$R_{b q} ^{\mathrm {reco}} $$ distribution.

**Table 3:** The measured values of $m_{\mathrm {top}}$ and the contributions of various sources to the uncertainty in the $t\bar{t}\rightarrow \text{ lepton+jets }$ and the $t\bar{t}\rightarrow \text{ dilepton }$ analyses. The corresponding uncertainties in the measured values of the $\text{ JSF }$ and $\text{ bJSF }$ are also shown for the $t\bar{t}\rightarrow \text{ lepton+jets }$ analysis. The statistical uncertainties associated with these values are typically 0.001 or smaller. The result of the $m_{\mathrm {top}}$ combination is shown in the rightmost columns, together with the correlation ($\rho$) within each uncertainty group as described in Sect. 8. The symbol n/a stands for not applicable. Values quoted as 0.00 are smaller than 0.005. Finally, the last line refers to the sum in quadrature of the statistical and systematic uncertainty components

Results	$t\bar{t}\rightarrow \text{ lepton+jets }$			$t\bar{t}\rightarrow \text{ dilepton }$	Combination	
	${m_{\mathrm {top}}^{\ell \mathrm {+jets}}}$ [${\mathrm { GeV}}$]	$\text{ JSF }$	$\text{ bJSF }$	${m_{\mathrm {top}}^{\mathrm {dil}}}$ [${\mathrm { GeV}}$]	$m_{\mathrm {top}}^{\mathrm {comb}}$ [${\mathrm { GeV}}$]	$\rho$
	172.33	1.019	1.003	173.79	172.99	
Statistics	0.75	0.003	0.008	0.54	0.48	0
Stat. comp. ($m_{\mathrm {top}}$)	0.23	n/a	n/a	0.54		
Stat. comp. ($\text{ JSF }$)	0.25	0.003	n/a	n/a		
Stat. comp. ($\text{ bJSF }$)	0.67	0.000	0.008	n/a		
Method	0.11 $\pm$ 0.10	0.001	0.001	0.09 $\pm$ 0.07	0.07	0
Signal MC	0.22 $\pm$ 0.21	0.004	0.002	0.26 $\pm$ 0.16	0.24	$+1.00$
Hadronisation	0.18 $\pm$ 0.12	0.007	0.013	0.53 $\pm$ 0.09	0.34	$+1.00$
ISR/FSR	0.32 $\pm$ 0.06	0.017	0.007	0.47 $\pm$ 0.05	0.04	$-1.00$
Underlying event	0.15 $\pm$ 0.07	0.001	0.003	0.05 $\pm$ 0.05	0.06	$-1.00$
Colour reconnection	0.11 $\pm$ 0.07	0.001	0.002	0.14 $\pm$ 0.05	0.01	$-1.00$
PDF	0.25 $\pm$ 0.00	0.001	0.002	0.11 $\pm$ 0.00	0.17	$+0.57$
$W/Z+$jets norm	0.02 $\pm$ 0.00	0.000	0.000	0.01 $\pm$ 0.00	0.02	$+1.00$
$W/Z+$jets shape	0.29 $\pm$ 0.00	0.000	0.004	0.00 $\pm$ 0.00	0.16	0
NP/fake-lepton norm.	0.10 $\pm$ 0.00	0.000	0.001	0.04 $\pm$ 0.00	0.07	$+1.00$
NP/fake-lepton shape	0.05 $\pm$ 0.00	0.000	0.001	0.01 $\pm$ 0.00	0.03	$+0.23$
Jet energy scale	0.58 $\pm$ 0.11	0.018	0.009	0.75 $\pm$ 0.08	0.41	$-0.23$
*b*-Jet energy scale	0.06 $\pm$ 0.03	0.000	0.010	0.68 $\pm$ 0.02	0.34	$+1.00$
Jet resolution	0.22 $\pm$ 0.11	0.007	0.001	0.19 $\pm$ 0.04	0.03	$-1.00$
Jet efficiency	0.12 $\pm$ 0.00	0.000	0.002	0.07 $\pm$ 0.00	0.10	$+1.00$
Jet vertex fraction	0.01 $\pm$ 0.00	0.000	0.000	0.00 $\pm$ 0.00	0.00	$-1.00$
$b\text{-tagging }$	0.50 $\pm$ 0.00	0.001	0.007	0.07 $\pm$ 0.00	0.25	$-0.77$
$E_{\text {T}}^{\text {miss}}$	0.15 $\pm$ 0.04	0.000	0.001	0.04 $\pm$ 0.03	0.08	$-0.15$
Leptons	0.04 $\pm$ 0.00	0.001	0.001	0.13 $\pm$ 0.00	0.05	$-0.34$
Pile-up	0.02 $\pm$ 0.01	0.000	0.000	0.01 $\pm$ 0.00	0.01	0
Total	1.27 $\pm$ 0.33	0.027	0.024	1.41 $\pm$ 0.24	0.91	$-0.07$

#### Lepton momentum and missing transverse momentum

The lepton momentum and the $E_{\text {T}}^{\text {miss}}$ are used in the event selection and reconstruction. For the leptons, the momentum scale, resolution and identification efficiency are measured using high-purity $Z\rightarrow \ell \ell$ data [60, 60]. The uncertainty due to any possible miscalibration is propagated to the analyses by changing the measured reconstruction efficiency, lepton $p_{\text {T}}$, and the corresponding resolution, within uncertainties.

The uncertainties from the energy scale and resolution corrections for leptons and jets are propagated to the $E_{\text {T}}^{\text {miss}}$. The systematic uncertainty related to the $E_{\text {T}}^{\text {miss}}$ accounts for uncertainties in the energies of calorimeter cells not associated with the reconstructed objects, and from cells associated with low-$p_{\text {T}}$ jets (7 GeV$< p_{\text {T}} <$ 20 GeV), as well as for the dependence of their energy on the number of pile-up interactions [60].

#### Pile-up

The residual systematic uncertainty due to pile-up was assessed by determining the dependence of the fitted top quark mass on the amount of pile-up activity, combined with uncertainties in modelling the amount of pile-up in the sample.

### Summary

The resulting sizes of all uncertainties and their sum in quadrature are given in Table 3. The total uncertainties on $${m_{\mathrm {top}}^{\ell \mathrm {+jets}}} $$, $$\text{ JSF } $$, $$\text{ bJSF } $$ and $${m_{\mathrm {top}}^{\mathrm {dil}}} $$, amount to $${1.27} {\mathrm { GeV}}$$, $${0.027} $$, $${0.024} $$ and $${1.41} {\mathrm { GeV}}$$, respectively. Within uncertainties, the fitted values of $$\text{ JSF } $$ and $$\text{ bJSF } $$ are consistent with unity.

## Combination of the $m_{\mathrm {top}}$ results

The results of the $t\bar{t}\rightarrow \text{ lepton+jets }$ and $t\bar{t}\rightarrow \text{ dilepton }$ analyses listed in Table 3 are combined using the Best Linear Unbiased Estimate (BLUE) method [75, 76], implemented as described in Refs. [77, 78]. The BLUE method determines the coefficients (weights) to be used in a linear combination of the input measurements by minimising the total uncertainty of the combined result. In the algorithm, both the statistical and systematic uncertainties, and the correlations ($\rho$) of the measurements, are taken into account, while assuming that all uncertainties are distributed according to Gaussian probability density functions.

### Correlation of the $t\bar{t}\rightarrow \text{ lepton+jets }$ and $t\bar{t}\rightarrow \text{ dilepton }$ measurements

To perform the combination, for each source of systematic uncertainty, the uncertainties as well as the correlation of the measurements of $m_{\mathrm {top}}$ were evaluated.

The measurements are taken as uncorrelated for the statistical, the method calibration and the pile-up uncertainties. For the remaining uncertainty components there are two possible situations. Either the measurements are fully correlated, $\rho =+1$, i.e. a simultaneous upward variation of the systematic uncertainty results in a positive (or negative) shift of $m_{\mathrm {top}}$ for both measurements, or fully anti-correlated, $\rho =-1$. In the latter case one measurement exhibits a positive shift and the other a negative one.

**Fig. 6:**
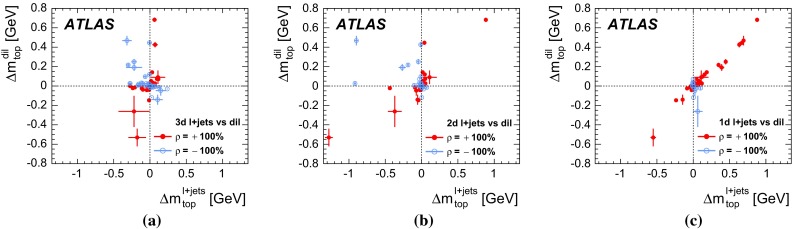
The systematic uncertainties of $m_{\mathrm {top}}$ in the $\ell \text{+jets }$ analysis versus those of the $\text{ dilepton }$ analysis. Figures **a**–**c** refer to the results evaluated for the three-dimensional analysis  (3d), two-dimensional analysis  (2d) and one-dimensional analysis  (1d). The *points* show the estimated systematic uncertainties on $m_{\mathrm {top}}$ for the two analyses, and the uncertainty bars reflect the corresponding statistical uncertainties. The *different colours* reflect the different correlations described in Sect. 8.1

Figure 6a shows the two dimensional distribution of the systematic uncertainties, denoted by ${\Delta {m_{\mathrm {top}}^{\ell \mathrm {+jets}}}}$ and ${\Delta {m_{\mathrm {top}}^{\mathrm {dil}}}}$, obtained in the $\ell \text{+jets }$ and $\text{ dilepton }$ analyses for all components of the sources of systematic uncertainty for which the measurements are correlated. The points show the estimated size of the uncertainties, and the error bars represent the statistical uncertainties on the estimates. Some uncertainty sources in Table 3, such as the uncertainty related to the choice of MC generator for signal events, contain only a single component. For these type of sources, the correlation is either $\rho =+1$ (red points) or $\rho =-1$ (blue points). The size of the uncertainty bars in Fig. 6a indicates that the distinction between $\rho =+1$ and $\rho =-1$ can be unambiguously made for all components that significantly contribute to the systematic uncertainty on $m_{\mathrm {top}}$.

For uncertainty sources that contain multiple components such as the JES uncertainty described in Appendix A, the correlations given in Table 3 differ from $\rho =\pm 1$. For these cases the correlation is obtained by adding the corresponding covariance terms of the components and dividing by the respective total uncertainties of the source.

For each systematic uncertainty, the size of ${\Delta {m_{\mathrm {top}}^{\ell \mathrm {+jets}}}}$ and ${\Delta {m_{\mathrm {top}}^{\mathrm {dil}}}}$, and the correlation of the measurements depend on the details of the analyses. This can be seen from Fig. 6b and c where the same information as in Fig. 6a is shown, but for different implementations of the $\ell \text{+jets }$ analysis, while leaving the $\text{ dilepton }$ analysis unchanged. Figure 6b corresponds to a two-dimensional analysis, similar to Ref. [8], which is realised by fixing the $\text{ bJSF }$ to unity. Finally, Fig. 6c shows the result of a one-dimensional analysis, in which the values of the $\text{ JSF }$ and $\text{ bJSF }$ are fixed to unity. For this implementation, as for the $\text{ dilepton }$ analysis, only $m_{\mathrm {top}}$ is obtained from the fit to data. Compared to the two-dimensional analysis, the three-dimensional analysis reduces some sources of uncertainty on $m_{\mathrm {top}}$. As an example, the rightmost red point in Fig. 6b, which corresponds to the bJES uncertainty, lies close to the vertical line in Fig. 6a, i.e. for the $\ell \text{+jets }$ analysis the impact of this source was considerably reduced by the $\text{ bJSF }$ determination from data. The change in the correlations of the measurements for specific sources of uncertainty, caused by a variation of the analysis strategy, is apparent from Fig. 6c, where for both analyses only $m_{\mathrm {top}}$ is obtained from the data. In this case the exploited observables are much more similar and consequently, the measurements of $m_{\mathrm {top}}$ are fully correlated for all sources of uncertainty that significantly contribute to the total uncertainty. This demonstrates that the three-dimensional analysis not only reduces the impact of some sources of uncertainty, mainly the JES and bJES uncertainties, but also makes the two measurements less correlated, thus increasing the gain in the combination of the two estimates of $m_{\mathrm {top}}$.

To best profit from the combination of the two measurements, their correlation should be as small as possible, see Ref. [78]. Consequently, the jet energy scale factors measured in the $\ell \text{+jets }$ analysis have not been propagated to the dilepton analysis, as was first done in Ref. [79]. Transferring the scales would require adding an additional systematic uncertainty to the $\text{ dilepton }$ analysis to account for the different jet energy scale factors caused by different kinematical selections and jet topologies of the two analyses. The two final states contain either two or four jets that have different distributions in jet $p_{\text {T}}$, and different amounts of final state QCD radiation. Most notably, this would also result in a large correlation of the measurements, similar to that observed for the one-dimensional analyses shown in Fig. 6c. Consequently, the knowledge of $m_{\mathrm {top}}$ from the $\ell \text{+jets }$ analysis would not significantly improve when including a $\text{ dilepton }$ measurement obtained with transferred jet energy scales. For an example of such a situation see Table VI of Ref. [79].

Using the correlations determined above, the combination of the $m_{\mathrm {top}}$ results of the $t\bar{t}\rightarrow \text{ lepton+jets }$ and $t\bar{t}\rightarrow \text{ dilepton }$ analyses yields:

$$\begin{aligned} m_{\mathrm {top}}^{\mathrm {comb}} &=  172.99 \pm 0.48~(\mathrm stat) \pm 0.78~(\mathrm syst) ~{\mathrm { GeV}}\\ &=  172.99 \pm 0.91 ~{\mathrm { GeV}}. \end{aligned}$$

This value corresponds to a $28\,\%$ gain in precision with respect to the more precise $\ell \text{+jets }$ measurement. The compatibility of the input measurements is very good, and corresponds to $0.75\sigma$ (${m_{\mathrm {top}}^{\ell \mathrm {+jets}}}-{m_{\mathrm {top}}^{\mathrm {dil}}} = -1.47 \pm 1.96~{\mathrm { GeV}}$). The BLUE weights of the results of the $t\bar{t}\rightarrow \text{ lepton+jets }$ and $t\bar{t}\rightarrow \text{ dilepton }$ analyses are 54.8 % and 45.2 %, respectively. The total correlation of the input measurements is $-7~\%$ and the $\chi ^2$ probability of the combination is 45.5 %. The list of all uncertainties of the combined result, together with the correlation of the measurements for each group of uncertainties, is provided in Table 3. The current precision is mostly limited by systematic uncertainties related to the MC modelling of $t\bar{t}$ events, and to the calibration of the jet energy scales.

### Stability of the results

The dependence of the combined result on the statistical uncertainties of the evaluated systematic uncertainties is investigated by performing one thousand BLUE combinations in which all input uncertainties are independently smeared using Gaussian functions centred at the expected values, and with a width corresponding to their statistical uncertainties. Using the smeared uncertainties, the correlations are re-evaluated for each pseudo-experiment. The combined $m_{\mathrm {top}}$ and its total uncertainty are distributed according to Gaussian functions of width 37  MeV and 43  MeV, respectively. Similarly, the BLUE combination weights and the total correlation are Gaussian distributed, with widths of 2.5 $\%$ and 6.1 $\%$, respectively. These effects are found to be negligible compared to the total uncertainty of the combined result. Consequently, no additional systematic uncertainty is assigned.

## Conclusion

The top quark mass was measured via a three-dimensional template method in the $$t\bar{t}\rightarrow \text{ lepton+jets } $$ final state, and using a one-dimensional template method in the $$t\bar{t}\rightarrow \text{ dilepton } $$ channel. Both analyses are based on $$\sqrt{s}=7$$ $${\mathrm { TeV}}$$ proton–proton collision ATLAS data from the 2011 LHC run corresponding to an integrated luminosity of $$4.6 $$ $$\text{ fb }^{-1}$$. In the $$\ell \text{+jets } $$ analysis, $$m_{\mathrm {top}} $$ is determined together with a global jet energy scale factor ($$\text{ JSF } $$) and a residual *b*-to-light-jet energy scale factor ($$\text{ bJSF } $$). The measured values are:

$$\begin{aligned} {m_{\mathrm {top}}^{\ell \mathrm {+jets}}}= & {} {172.33} \pm {0.75} ~\mathrm {(stat + \text{ JSF } + \text{ bJSF })} \pm {1.02} ~\mathrm {(syst)}~{\mathrm { GeV}}, \\ \text{ JSF }= & {} {{1.019} \,\pm {0.003} ~\mathrm {(stat)}\,\pm {0.027} ~\mathrm {(syst)}},\\ \text{ bJSF }= & {} {{1.003} \,\pm {0.008} ~\mathrm {(stat)}\,\pm {0.023} ~\mathrm {(syst)}}, \\ {m_{\mathrm {top}}^{\mathrm {dil}}}= & {} {173.79} \pm {0.54} ~\mathrm {(stat)} \pm {1.30} ~\mathrm {(syst)}~{\mathrm { GeV}}. \end{aligned}$$

These measurements are consistent with the ATLAS measurement in the fully hadronic decay channel [13], and supersede the previous result described in Ref. [8].

A combination of the $t\bar{t}\rightarrow \text{ lepton+jets }$ and $t\bar{t}\rightarrow \text{ dilepton }$ results is performed using the BLUE technique, exploiting the full uncertainty breakdown, and taking into account the correlation of the measurements for all sources of the systematic uncertainty. The result is:

$$\begin{aligned} m_{\mathrm {top}}^{\mathrm {comb}} &= 172.99 \pm 0.48~(\mathrm stat) \pm 0.78~(\mathrm syst)\, {\mathrm { GeV}} \\ &= 172.99 \pm 0.91 \, {\mathrm { GeV}}. \end{aligned}$$

This corresponds to a gain in precision with respect to the more precise $\ell \text{+jets }$ measurement of $28\, \%$. The total uncertainty of the combination corresponds to 0.91 ${\mathrm { GeV}}$ and is currently dominated by systematic uncertainties due to jet calibration and modelling of the $t\bar{t}$ events.

## Appendix A: Jet energy scale uncertainty: detailed components

The relative JES uncertainty varies from about 1 % to 3 % depending on jet properties as given in Section 13 of Ref. [58]. These components correspond to the eigenvectors of the reduced covariance matrix for the JES uncertainties, as described in Section 13.3 of Ref. [58]. The initial sources of nuisance parameters (NP) originating from the in-situ determination of the JES are listed in Table 10 of Ref. [58]. According to their nature, they are categorised into the classes: detector description, physics modelling, statistics and method, mixed detector and modelling. Finally, following Section 13.6 of Ref. [58], a reduction of the number of nuisance parameters is performed for each category giving various components. Their $p_{\text {T}}$ dependences are given in Fig. 42 of Ref. [58]. The total JES uncertainty is provided together with its 21 sub-components in Table 4. Their separate effects on the fitted top quark mass are summed in quadrature to determine the total jet energy scale uncertainty given in Table 3. For further details about each component, see Ref. [58].

**Table 4:** The individual components of the JES uncertainty according to Ref. [58], together with the corresponding uncertainties on ${m_{\mathrm {top}}^{\ell \mathrm {+jets}}}$, $\text{ JSF }$, $\text{ bJSF }$, ${m_{\mathrm {top}}^{\mathrm {dil}}}$, and $m_{\mathrm {top}}^{\mathrm {comb}}$. Some components listed are calculated as the sum in quadrature of several sub-components. The corresponding measurement correlations per group described in Sect. 8 are also reported

	$t\bar{t}\rightarrow \text{ lepton+jets }$			$t\bar{t}\rightarrow \text{ dilepton }$	Combination	
	$\Delta {m_{\mathrm {top}}^{\ell \mathrm {+jets}}}$ [${\mathrm { GeV}}$]	$\Delta \text{ JSF }$	$\Delta \text{ bJSF }$	$\Delta {m_{\mathrm {top}}^{\mathrm {dil}}}$ [${\mathrm { GeV}}$]	$\Delta m_{\mathrm {top}}^{\mathrm {comb}}$ [${\mathrm { GeV}}$]	$\rho$
Statistical (total)	$0.18 \pm 0.04$	0.003	0.001	$0.16 \pm 0.03$	0.11	$-0.25$
Statistical NP1	$-0.17 \pm 0.02$	$+0.002$	$+0.001$	$+0.01 \pm 0.02$	0.09	$-1.00$
Statistical NP2	$+0.02 \pm 0.00$	$+0.001$	$-0.000$	$+0.05 \pm 0.00$	0.03	$+1.00$
Statistical NP3	$-0.01 \pm 0.02$	$+0.001$	$+0.001$	$+0.12 \pm 0.02$	0.05	$-1.00$
$\eta$ inter-calibration (stat.)	$-0.07 \pm 0.02$	$+0.001$	$+0.001$	$+0.10 \pm 0.02$	0.01	$-1.00$
Modelling (total)	$0.31 \pm 0.06$	0.009	0.002	$0.52 \pm 0.04$	0.26	$-0.18$
Modelling NP1	$-0.30 \pm 0.03$	$+0.006$	$+0.001$	$+0.22 \pm 0.02$	0.07	$-1.00$
Modelling NP2	$+0.03 \pm 0.02$	$+0.002$	$-0.000$	$+0.14 \pm 0.02$	0.08	$+1.00$
Modelling NP3	$-0.01 \pm 0.02$	$-0.002$	$-0.000$	$-0.15 \pm 0.02$	0.07	$+1.00$
Modelling NP4	$-0.01 \pm 0.00$	$+0.000$	$+0.000$	$+0.02 \pm 0.00$	0.00	$-1.00$
$\eta$ inter-calibration (model)	$+0.07 \pm 0.04$	$+0.007$	$-0.001$	$+0.43 \pm 0.03$	0.23	$+1.00$
Detector (total)	$0.05 \pm 0.03$	0.007	0.001	$0.45 \pm 0.04$	0.20	$-0.19$
Detector NP1	$-0.01 \pm 0.03$	$+0.007$	$+0.001$	$+0.45 \pm 0.02$	0.20	$-1.00$
Detector NP2	$-0.05 \pm 0.00$	$+0.000$	$+0.001$	$+0.03 \pm 0.00$	0.02	$-1.00$
Mixed (total)	$0.02 \pm 0.02$	0.001	0.001	$0.03 \pm 0.02$	0.01	$-0.80$
Mixed NP1	$-0.02 \pm 0.00$	$+0.000$	$+0.001$	$+0.02 \pm 0.00$	0.00	$-1.00$
Mixed NP2	$+0.00 \pm 0.02$	$+0.001$	$-0.000$	$+0.02 \pm 0.02$	0.01	$+1.00$
Single particle high-$p_{\text {T}}$	$+0.00 \pm 0.00$	$+0.000$	$-0.000$	$+0.00 \pm 0.00$	0.00	$+1.00$
Relative non-closure MC	$+0.00 \pm 0.02$	$+0.001$	$-0.000$	$+0.03 \pm 0.02$	0.02	$+1.00$
Pile-up (total)	$0.15 \pm 0.04$	0.001	0.002	$0.04 \pm 0.03$	0.09	$+0.03$
Pile-up: Offset($\mu$)	$-0.11 \pm 0.02$	$-0.001$	$+0.001$	$-0.02 \pm 0.02$	0.07	$+1.00$
Pile-up: Offset($n_\mathrm{vtx}$)	$-0.10 \pm 0.04$	$-0.000$	$+0.001$	$+0.03 \pm 0.03$	0.04	$-1.00$
Flavour (total)	$0.36 \pm 0.04$	0.012	0.008	$0.03 \pm 0.03$	0.20	$-0.17$
Flavour composition	$-0.24 \pm 0.02$	$+0.006$	$-0.002$	$-0.02 \pm 0.02$	0.14	$+1.00$
Flavour response	$-0.28 \pm 0.03$	$+0.011$	$-0.008$	$+0.03 \pm 0.02$	0.14	$-1.00$
Close-by jets	$-0.22 \pm 0.04$	$+0.005$	$+0.002$	$+0.25 \pm 0.03$	0.01	$-1.00$
*b*-Jet energy scale	$+0.06 \pm 0.03$	$+0.000$	$+0.010$	$+0.68 \pm 0.02$	0.34	$+1.00$
Total (without bJES)	0.58 $\pm$ 0.11	0.018	0.009	0.75 $\pm$ 0.08	0.41	$-0.23$

## Footnotes

1 ATLAS uses a right-handed coordinate system with its origin at the nominal interaction point (IP) in the centre of the detector and the *z*-axis along the beam pipe. The *x*-axis points from the IP to the centre of the LHC ring, and the *y*-axis points upwards. Cylindrical coordinates $(r,\phi )$ are used in the transverse plane, $\phi$ being the azimuthal angle around the beam pipe. The pseudorapidity is defined in terms of the polar angle $\theta$ as $\eta = -\ln \tan (\theta /2)$. Angular distances are defined as $\Delta R \equiv \sqrt{(\Delta \eta )^{2} + (\Delta \phi )^{2}}$.

2 Here $$m_{\mathrm {T}}^{W} $$ is the $$W $$ boson transverse mass, defined as $$\sqrt{2\,p_\mathrm {T,\ell }\,p_\mathrm {T,\nu }[1-\cos (\phi _{\ell }-\phi _{\nu })]}$$, where the measured $$E_{\text {T}}^{\text {miss}} $$ vector provides the neutrino ($$\nu $$) information.
